# Stereotypically thinking: Norms for stereotypical gender nouns in Polish and English

**DOI:** 10.1371/journal.pone.0312405

**Published:** 2024-12-31

**Authors:** Katarzyna Jankowiak, Marcin Naranowicz, Anna Skałba, Dariusz Drążkowski, Joanna Pawelczyk

**Affiliations:** 1 Faculty of English, Adam Mickiewicz University, Poznań, Poland; 2 Faculty of Psychology and Cognitive Sciences, Adam Mickiewicz University, Poznań, Poland; University of Minho School of Psychology: Universidade do Minho Escola de Psicologia, PORTUGAL

## Abstract

The present contribution provides norms for a database of Polish (a grammatical gender language) and English (a natural gender language) stereotypical gender and neutral nouns. A total of 317 participants rated the degree of stereotypically feminine and masculine features when presented with 240 nouns in each language. The stimuli were highly controlled for a number of psycholinguistic variables, including word frequency, the number of letters and syllables, age of acquisition, concreteness, valence, and arousal. The results of the ratings revealed that gender stereotypical features were observed in both language systems, thus suggesting that single words that do not explicitly reference any male or female characteristics can activate stereotypically feminine and masculine schemas, regardless of grammatical gender. Furthermore, the results suggested a stronger internalization of gender stereotypes among female relative to male participants as well as among sex-typed individuals, therefore pointing to the crucial role of gender and gender schema in how sensitive individuals are to gender stereotypical attributes. The norms reported in the present article aim to broaden researchers’ stimulus choices and allow for consistency across different laboratories and research projects on gender stereotype processing. The adaptation of this database to other languages or cultures could also enable a cross-cultural comparison of empirical findings on stereotype processing.

## Introduction

The bidirectional relationship between language and social cognition has been long established and supported by research, showing that language both affects and is affected by our perceptual processes [1, 2]. Such a relationship is especially evident when studying gender stereotypes, since language, as “the major symbolic system of human species” [3], encodes stereotypical beliefs and assumptions, and functions as a powerful resource in accumulating and further transmitting that knowledge [4]. Gender stereotypes, being differently reflected in different languages, display culture-specific beliefs about the characteristics of men and women, and impact how we both experience and interpret information concerning them as well as how we judge their abilities and habits [5–7]. Previous research on stereotypical gender nouns (e.g., nouns that stereotypically, yet not explicitly, activate a male or female referent; *a doll; a car*) has repeatedly shown that gender stereotypes are deeply rooted in language (e.g., [1, 8–11]) and might be even processed somewhat similar to definitional gender nouns (e.g., *a girl*; *a king* [12, 13]). However, little scholarly attention has been devoted to examining whether such a perception might be modulated by a language system (For an overview of how grammatical gender is manifested across different languages, see [14].). In the present contribution, we provide the normed dataset of single stereotypical gender nouns that carry gendered (i.e., feminine or masculine), as compared to neutral, associations, and test if these are stronger in a grammatical gender (i.e., Polish) relative to a natural gender (i.e., English) language. Furthermore, we provide insights into whether and to what extent individuals’ sensitivity to gender stereotypical attributes might be modulated by their gender and gender schema.

Gender stereotypes are able to “actively influence people’s perception and evaluation of interlocutors” [4], and their power can indeed be evident in people’s attribution of feminine or masculine characteristics to non-gendered things. As the most salient social category, gender is often described as omni-relevant [15, 16]. In situations where the information about the gender of an individual is not accessible, we tend to rely on other symbolic cues that may index a person’s gender. Similarly, various aspects of our social life (e.g., activities, the way we use language, etc.), although not directly referencing gender, may indirectly index femininity or masculinity (see also [3]). Put differently, ‘things’ tend to be perceived/read/interpreted in terms of gender binarism (i.e., as feminine or masculine), and therefore, “whatever we [do or] say is gendered” [17] (We might, however, start questioning this claim, since increasingly often the binarism of gender is being challenged and/or contested. This, in turn, indicates a move away from perceiving and interpreting our social life as gendered.).

Since gender functions as a salient social category, naturally gender stereotypes are correspondingly strong. In sociological and psychological literature, women are described as communal and other-centered [18], while men as agentic [19, 20] (see [4]). Since men and women are positioned as socially relational categories, they are expected to occupy different social roles and positions. Whereas women are believed to be warm/emotional and nurturing (building communality), men are ascribed competence and independence (building agency; see [21]).

The content of gender stereotypes is to a great extent recognized globally, as there are common social expectations as to what men and women are (to be) like. It is interesting, however, to consider whether the users of particular languages (e.g., users of grammatical gender vs. natural gender languages) perceive nouns differently in terms of their potential gendered load. Namely, it has been shown that *grammatical gender* languages (i.e., languages in which each noun is marked for gender; e.g., German, Polish, and Spanish) elicit a more robust activation of gender stereotypes as compared to *natural gender* languages (e.g., English and Scandinavian languages), where feminine and masculine gender is not distinguished [22–24]. Specifically, natural gender languages have been suggested to increase the cognitive availability of mental representations of female exemplars as compared to masculine generics [25], therefore potentially reducing the male bias in mental representations [26, 27]. In line with these findings, cross-country analyses have shown that in countries where gendered languages are spoken, there is less gender equality [24], educational attainment and female labor force participation are lower [28], and intimate partner violence is seen as more justifiable [29] compared to countries with other grammatical gender systems.

The present studies focus on two languages–Polish and English, which differ with respect to gender marking on nouns. In English (i.e., a natural gender language), nouns do not have grammatical marking of gender, and they “are gendered according to the biological sex of their referents” [10]. On the other hand, in Polish (i.e., a grammatical gender language), nouns always carry a grammatical gender marking. Through grammatical agreement, gender marking on nouns in Polish needs to be consistent with other speech parts (“The Polish language has five gender classes in the singular (masculine personal, masculine animate, masculine inanimate, neuter and feminine). In the plural, with respect to syntactic concord, a gender distinction is made between masculine personal (Polish *męskoosobowy*) and nonmasculine personal (Polish *niemęskoosobowy*), which includes female human, animate nonhuman, and nonanimate referents. Gender is marked throughout the noun phrase and many verb phrases. The relationship between grammatical gender and the sex of the referent is symmetrical in the singular, where mot animate female referents are marked for feminine gender and male referents for masculine gender. However, in the plural, the nonmasculine personal includes nouns whose animate referents as female as well as nouns referring to nonanimate referents.” (Kiełkiewicz-Janowiak and Pawelczyk 2014: 358).). Consequently, the relationship between grammatical and stereotypical gender is particularly complex in grammatically gender languages, given that grammatical gender is highly transparent and therefore provides a reliable cue during language processing. Indeed, the grammatical gender effect, which reflects the influence of grammatical gender on social cognition [30, 31], has been robustly identified in Polish [10, 32, 33]; yet, it remains under-investigated to whether such an effect can be extended to stereotypical gender nouns, and whether this can also be observed in a natural gender language, such as English. It has been recently suggested by Casado et al. [34] that though grammatical gender typically exerts a stronger influence on language processing due to its transparent cues, stereotypical gender can still play a significant role, especially when it conflicts with grammatical gender. Understanding how these two sources of information interact is crucial for a more comprehensive understanding of how gender stereotypes are represented in the mental lexicon.

Previous psycholinguistic research has explored the behavioral and electrophysiological correlates of processing words laden with gender stereotypes, showing that gender stereotype information is accessed highly automatically during visual word recognition [5, 6, 35, 36]. Stereotypical gender associations are often a reflection of societal norms embedded in language [37], and the activation of these stereotypes influences both language comprehension and production [38, 39]. For example, Pesciarelli et al. [5] presented participants with feminine or masculine third-person singular pronouns (i.e., she/he) preceded by stereotypically gendered prime words (e.g., teacher/female, driver/male). Their results revealed that processing stereotypically incongruent pairs (e.g., teacher–he) imposed greater cognitive demands than congruent pairs (e.g., teacher–she). This suggests that stereotype mismatch triggers additional cognitive effort. Moreover, other findings show that grammatical gender can bias perception, leading speakers to attribute stereotypically masculine or feminine traits to inanimate objects based solely on their grammatical gender [30]. Such research supports the immediacy hypothesis [40, 41], indicating that stereotypical gender is incorporated into the meaning representation of words or sentences as soon as it becomes available [5]. In sum, psycholinguistic research highlights that gender stereotypes are not only automatically activated but also embedded in the mental representation of words and sentences. These stereotypes influence both behavioral and neural responses to language. The automatic activation of gender stereotypes can affect word processing efficiency and has broader implications for how language perpetuates or challenges societal gender norms.

Interestingly, previous research has also reported differences in how males as compared to females perceive stereotypically masculine and feminine features, with females being assumed to be more sensitive to gender differences relative to males (e.g., [42, 43]; but see [44]). Such patterns might result from a fundamental role gender stereotypes play as far as the barriers for females’ advancements are concerned [45–47]. This seems to be in line with recent research suggesting that females characterize themselves in more gender stereotypical terms [48], demonstrating a stronger implicit gender stereotypical bias relative to males [49]. Such an increased internalization of gender stereotypes in females relative to males is also likely to be reflected in how female individuals perceive gender stereotypes in language, where females are expected to show a more pronounced sensitivity to identifying gender features within single nouns.

In addition to participants’ gender, their perception of stereotypical attributes might be modulated by their individual stereotypically feminine or masculine traits. In line with the gender schema theory [50], individuals are inclined to organize, perceive, process, select, and memorize sex-typed information based on their own gender schema, which “becomes a prescriptive standard or guide” for evaluating the self and others [50]. Particularly, sex-typed male and female individuals, who strongly adhere to their expected male and female gender roles, respectively, are assumed to be more predisposed to use gender schemas to organize and process sex-typed information about themselves and others. In contrast, those that either violate their expected gender roles, i.e., individuals that display traits of their opposite gender (i.e., cross-sex-typed individuals), those with high levels of both masculine and feminine traits (i.e., androgynous individuals), or those displaying low levels of both of those features (i.e., undifferentiated individuals), are more likely to showcase a decreased sensitivity to stereotypically masculine and feminine features [50]. Consequently, the perception of gender stereotypes encoded in language is assumed to be modulated by participants’ gender schema, with the highest sensitivity to stereotypical features observed in sex-typed individuals.

In the following sections we present our ratings for a database of Polish (Study I) and English (Study II) nouns, as assessed in terms of their stereotypically feminine or masculine load, as compared to the neutral (control) condition. The database aims to complement previously prepared corpora that provide words that were, however, not controlled for the part of speech [51] nor for variables such as word frequency, valence, concreteness, or the number of letters and syllables [52]. This lack of control made the corpora not sufficiently developed to be employed in well-controlled psycholinguistic experiments. Also, the available datasets do not allow for a cross-linguistic comparison, as they are mostly focused on English only [53] (see [54] for a discussion). In addition to compiling an extensive database of stereotypical gender nouns, we aim to provide insights into the level of stereotypicality of such nouns as potentially dependent on a language system (Polish: grammatical gender vs. English: natural gender languages), and to show whether and how respondents’ individual differences (i.e., gender and gender schema) modulate the sensitivity to gender stereotypical features. First, we expect gender stereotypes to be more ingrained in the nouns of the grammatical gender (i.e., Polish) as compared to the natural gender language (i.e., English). Second, we hypothesize that irrespective of the language system, female participants will showcase a more pronounced sensitivity to gender stereotypical attributes relative to men [42, 43], with the effect being potentially stronger in sex-typed individuals [50]. Also, given that previous research has pointed to valence-driven differences between stereotypically feminine and masculine words [55, 56], we exploratorily analyze the relationship between word valence and word femininity/masculinity.

## Study I: The perception of Polish nouns

### Methods

#### Participants

The original sample included 170 participants, 22 of whom were excluded from the analyses due to providing incomplete answers or following a response set. This resulted in the total sample of 148 participants (76 females, 72 males), who were all native speakers of Polish aged 18–40 (*M* = 25.15; 95% CI [24.41, 25.89]). As one of our research questions addresses the role of participants’ gender in stereotype perception, non-binary and transgender individuals were not qualified for participation. Additionally, we qualified only the individuals above the age of 18 and below the age of 40, so that our sample was relatively homogeneous in terms of how participants internalize gender stereotypes. Participants were mostly recruited from the sample of students and graduates of [intentionally removed to ensure anonymity], 50% of whom were undergraduate students, less than 20% were holders of a Bachelor’s degree, and about 25%–holders of a Master’s degree. In terms of their gender role identities, participants were classified as sex-typed (*n* = 48), cross-sex (*n* = 38), androgynous (*n* = 33), or undifferentiated (*n* = 29) individuals based on the Polish adaptation of the Bem Sex Role Inventory (BSRI) [57].

#### Stimuli

The stimuli used in the present study included single stereotypical gender nouns selected from the SUBTLEX-PL database [58]. First, we generated a list of nouns of Zipf frequency values between 1.5 and 5.5. Having excluded Polish–English cognates, polysemous words, as well as nouns directly referring to gender (i.e., definitional nouns, such as *a boy*; *a queen*), we divided the remaining items into three categories on the basis of their gender stereotypicality. We shortlisted 60 stereotypically feminine (e.g., *a doll*; *lips*), 60 stereotypically masculine (e.g., *a gun; an engine*), and 120 neutral nouns (e.g., *a trip*; *a crowd*), which were assigned to their respective conditions based on an internal rating conducted among three native speakers of Polish. The pre-selection of the stimuli was intended as an initial step to ensure that the words chosen were representative and relevant for the categories under study. To create a robust and highly controlled database of words suitable for experimental studies, we accounted for several psycholinguistic variables, including word frequency, number of letters and syllables, age of acquisition, concreteness, valence, and arousal. Controlling for these variables is standard practice in the creation of word databases (e.g., [59–61]), as they are known to significantly influence word processing, such as reaction times and event-related potentials. By managing these variables, we aim to ensure precise word selection, facilitating a wide range of experimental designs. Table 1 provides examples of the stimuli used. The selected nouns were matched across conditions for such psycholinguistic variables as frequency, valence, arousal, concreteness, age of acquisition, as well as number of letters and syllables. Table 2 presents ranges, means, and confidence intervals for all these variables.

**Table 1:** Examples of the stimuli used in Study I and Study II.

	Stereotypically feminine words	Stereotypically masculine words	Stereotypically neutral words
Polish (Study I)	ślub (Eng. wedding)	rząd (Eng. government)	klucz (Eng. key)
	taniec (Eng. dance)	wojsko (Eng. army)	walizka (Eng. suitcase)
	kąpiel (Eng. bath)	silnik (Eng. engine)	jezioro (Eng. lake)
	kwiat (Eng. flower)	kopalnia (Eng. mine)	śnieg (Eng. snow)
	kuchnia (Eng. kitchen)	piwo (Eng. beer)	drzewo (Eng. tree)
English (Study II)	fashion	muscle	bench
	lullaby	jailhouse	pumpkin
	family	factory	envelope
	dollhouse	hammer	spoon
	suntan	wallet	elbow

**Table 2:** Ranges and means (with 95% confidence intervals) for the psycholinguistic variables across the three word types for the Polish items.

	Range	Mean	Stereotypically feminine words (*n* = 60)	Stereotypically masculine words (*n* = 60)	Stereotypically neutral words (*n* = 120)
Frequency^1^	1.68–5.27	3.36 [3.27, 3.45]	3.23 [3.06, 3.40]	3.33 [3.13, 3.54]	3.45 [3.33, 3.56]
Valence^2^	2.08–7.66	5.18 [5.04, 5.32]	5.80 [5.60, 6.01]	4.65 [4.43, 4.88]	5.14 [4.93, 5.34]
Arousal^2^	2.64–7.14	3.77 [3.67, 3.88]	3.56 [3.41, 3.70]	4.18 [3.94, 4.42]	3.68 [3.54, 3.82]
Concreteness^3^	1.44–4.54	2.36 [2.26, 2.46]	2.19 [2.04, 2.34]	2.39 [2.20, 2.57]	2.43 [2.27, 2.58]
Age of acquisition^4^	5.31–12.51	8.21 [8.03, 8.39]	8.09 [7.75, 8.43]	9.06 [8.70, 9.42]	7.85 [7.62, 8.08]
Letters	4–12	6.33 [6.12, 6.54]	6.72 [6.32, 7.11]	6.20 [5.76, 6.64]	6.20 [5.90, 6.50]
Syllables	1–4	2.25 [2.16, 2.35]	2.38 [2.20, 2.57]	2.18 [1.99, 2.38]	2.26 [2.09, 2.36]

Scales and databases:

^1^Frequency: 1 (*low*)– 7 (*high*) scale, SUBTLEX-PL [58].

^2^Valence and Arousal: 1 (*negative/unarousing*)– 9 (*positive/arousing*) scales, ANPW–R [59].

^3^Concreteness: 1 (*concrete*)– 9 (*abstract*) scale, ANPW–R [59].

^4^Age of Acquisition: ANPW–R [59].

Given that Polish is a grammatical gender language, there was no correspondence between category classification and grammatical gender of the shortlisted nouns. However, controlling for this variable would not have been possible without compromising on other psycholinguistic variables and reaching the required numbers of words in each condition. Overall, almost half of the nouns were grammatically feminine (45%), with the proportions ranging from 55% in the feminine condition, to 43% in the masculine and 42% in the neuter one. Somewhat less numerous were grammatically masculine nouns (38%), with the highest percentage in the masculine condition (47%), followed by neuter (39%) and feminine (28%) ones. Neuter and *plurale tantum* nouns were in the minority, as their overall proportions corresponded to 12% and 4%, respectively.

For the surveys, the stimuli were divided into five lists, each including 90 randomly selected nouns, so that each survey consisted of 30 items in each of the three conditions (feminine, masculine, and neutral), thus making the distribution of the three conditions equal in each survey list. Each survey was completed by the minimum of 27–30 participants (13–15 males and 13–15 females).

#### Procedure

Ratings on both Polish (Study I) and English (Study II) stimuli were collected using online surveys (SurveyMonkey, Momentive Inc.). Participant recruitment took place between April 3^rd^ and June 22^nd^, 2022.

The present study was conducted as part of a larger research project, which was approved by the Ethics Committee for Research Involving Human Participants at Adam Mickiewicz University, Poznan (Resolution No. 12/2021/2022). Participants were recruited via social media platforms and Internet forums and were randomly assigned to one of the five survey lists. Having given their written informed consent to participate in the study, participants were asked to rate how stereotypically feminine and masculine the selected nouns were (i.e., the degree of their associations with the stereotypes assigned to females and males, respectively). Each word from a 90-item list was rated on two seven-point Likert scales: the femininity scale (1 –*very unfeminine*, 7 –*very feminine*) and the masculinity scale (1 –*very unmasculine*, 7 –*very masculine*). Providing rating on two scales separately instead of a single one including both femininity and masculinity (i.e., 1 –*very feminine*, 7 –*very masculine*, or vice versa) enabled for the collection of more fine-grained detail regarding the stereotypical load of the selected nouns. Importantly, these scales included a midpoint (4 = neither unfeminine nor feminine / neither masculine nor unmasculine), which allowed for neutral responses. By incorporating these midpoints, we aimed to capture a spectrum of gendered perceptions rather than forcing a binary categorization. This approach enabled us to assess the degrees of femininity and masculinity independently, thereby providing a more thorough understanding of how these traits are perceived. The order of the femininity and masculinity scales was randomized across participants, with the same nouns not repeated across the two scales in one survey list.

Then, participants provided basic sociodemographic information, including their age, gender, and the level of education. Finally, participants completed the BSRI [57], in which they rated on a seven-point Likert scale how much each of the 40 characteristics (10 feminine, 10 masculine, 20 neutral) corresponded to them (1 –*never or almost never true*, 7 –*always or almost always true*). The BSRI aims to assess feminine and masculine characteristics, which helps to determine the gender role with which the participant identifies. The completion of the whole survey took about 15 minutes.

#### Data analysis

All analyses were performed in R [62].

*Reliability of the ratings*. Inter-class correlation coefficients (ICC) were calculated using a two-way random-effect model based on the mean of multiple raters as a measure of inter-rater reliability. Also, Cronbach’s alpha coefficients were calculated to assess internal consistency of the femininity and masculinity scales across all surveys.

*Word femininity and masculinity ratings*. The femininity and masculinity ratings were analyzed separately using linear mixed-effects models (LMMs) [63–66] on the basis of 2 (Gender: Female vs. Male participants) × 3 (Word type: Stereotypically feminine vs. Stereotypically masculine vs. Neutral words) design, using the *lme4* package [67] for R [62]. Gender and Word type were between- and within-subject factors, respectively. A maximal model was first computed with a full random-effect structure, including participant-related and item-related variance components for intercepts and by-participant and by-item random slopes for fixed effects [65]. The controlled psycholinguistic variables (i.e., word frequency, valence, arousal, concreteness, age of acquisition, syllables, and letters) along with participants’ sociodemographic variables (i.e., the adopted gender roles) were exploratorily included in the models as covariates. When the data did not support the execution of the maximal model random structure, we reduced the model complexity to arrive at a parsimonious model [67]. To do so, we computed principal component analyses of the random structure and then kept the number of principal components that cumulatively accounted for 100% of the variance. *b* estimates and significance of fixed effects and interactions (*p*-values) were based on the Satterthwaite approximation for LMM (the *lmerTest* package) [68] for R [60]. *Post-hoc* analyses were calculated using the *emmeans* package [69] for R [62].

*The role of psycholinguistic variables in word ratings*. In order to exploratorily analyze the interplay between word valence and word femininity/masculinity ratings, we performed ordinal logistic regression analyses (i.e., cumulative link models) using the *ordinal* package [70], with femininity and masculinity ratings as ordinal dependent variables and valence as an independent variable. Additionally, to provide information on other variables controlled for when designing the stimuli (i.e., word frequency, arousal, concreteness, age of acquisition, the number of syllables and letters, and a grammatical gender form in the case of the Polish items), these independent psycholinguistic variables were also included in the analyses. The predictor variables were tested a priori to verify whether there was no violation of the assumption of multicollinearity. Pearson correlation coefficients (*r*) were then calculated to further explore the associations between the femininity as well as masculinity ratings and the psycholinguistic variables.

*The role of gender role identities in word ratings*. To better understand the role of gender role identities in word ratings, the femininity and masculinity ratings were also analyzed separately using LMMs (see above) on the basis of 3 (Word type: Stereotypically feminine vs. Stereotypically masculine vs. Neutral words) × 4 (Gender role identity: Sex-types vs. Cross-sex vs. Androgynous vs. Undifferentiated) design, with Word type as a within-subject factor and Gender role identity as a between-subject factor.

*The role of nouns’ grammatical gender in word ratings*. Additionally, since Polish represents a grammatical gender language, the femininity and masculinity ratings of the Polish items were also exploratorily analyzed using LMMs (see above) on the basis of 2 (Word type: Stereotypically feminine vs. Stereotypically masculine) × 2 (Grammatical gender: Grammatically feminine vs. Grammatically masculine) design.

### Results

#### Reliability of the ratings

**Fig 1:**
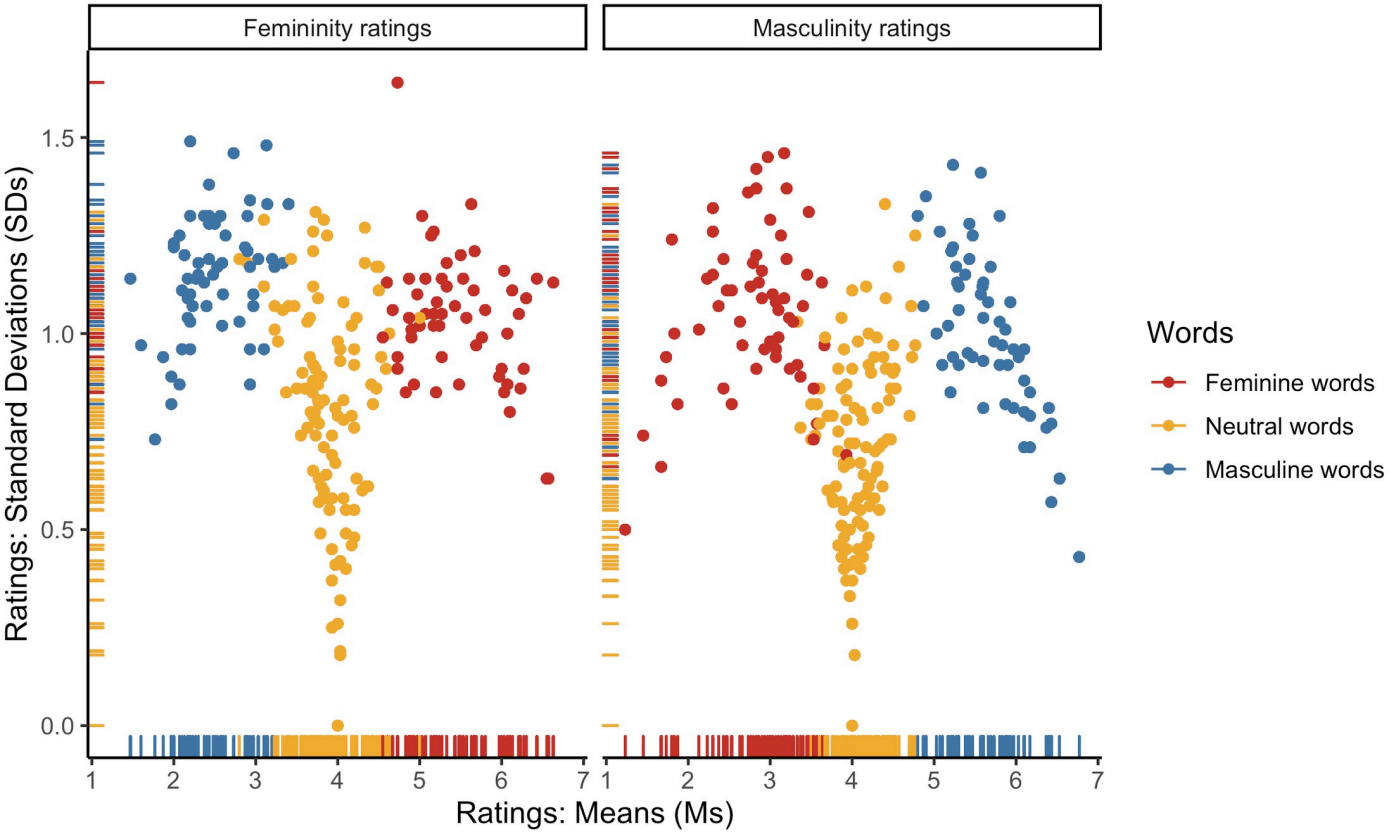
Distributions of rating means (Ms) and standard deviations (SDs) for the Polish items: (i) a left-biased distribution of the femininity ratings of the stereotypically masculine words and the masculinity ratings of the stereotypically feminine words; (ii) a right-biased distribution of the femininity ratings of the stereotypically feminine words and the masculinity ratings of the stereotypically masculine words; (iii) a center-biased distribution of the femininity and masculinity ratings of the neutral words.

**Fig 2:**
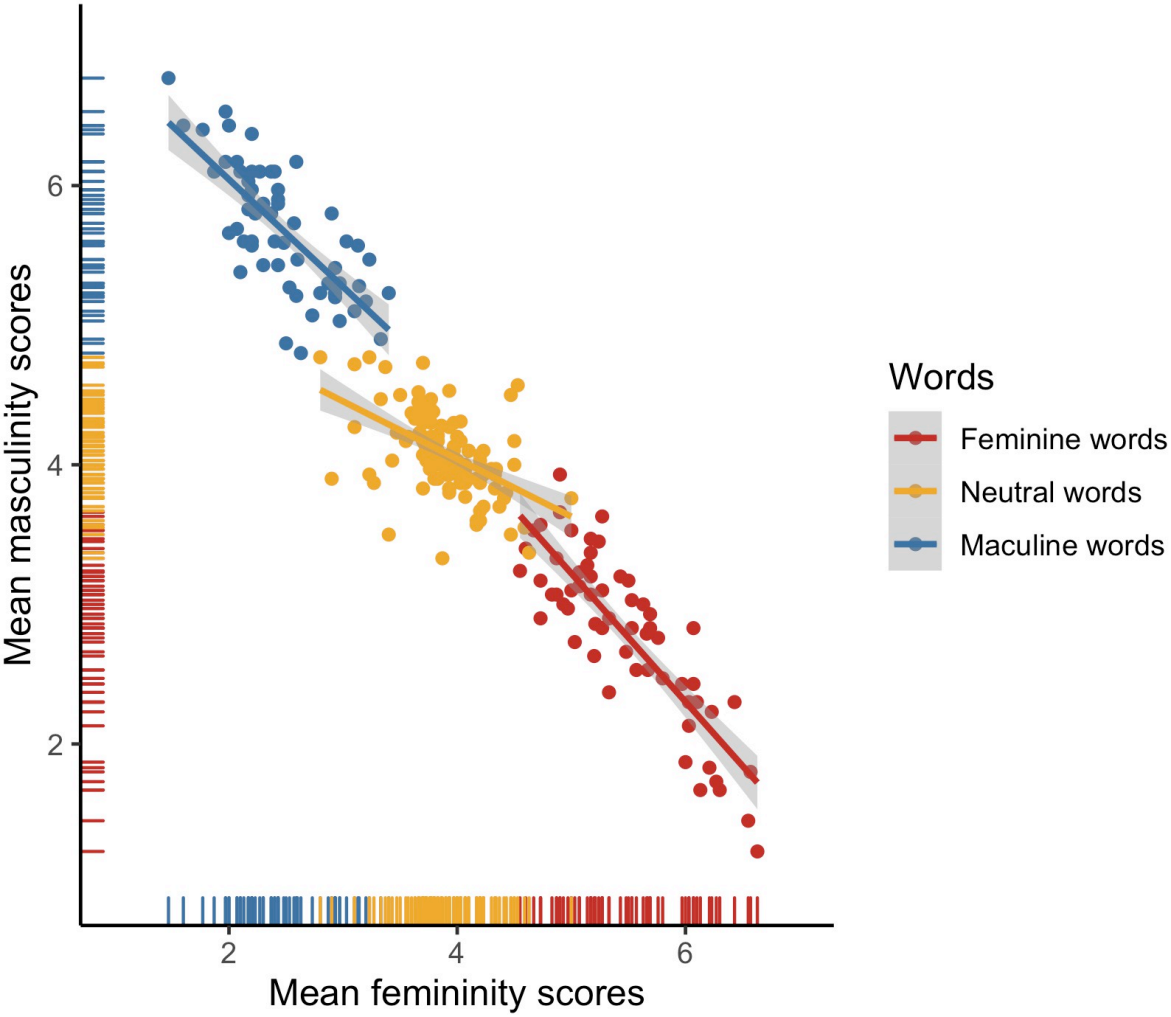
Associations between the mean masculinity and femininity scores for the Polish items.

ICCs pointed to high inter-rater reliability for both the femininity (ICC = .98) and masculinity ratings of Polish items (ICC = .98). Furthermore, both the femininity (*α =* .80) and masculinity (*α =* .76) scales demonstrated adequate degrees of internal consistency across all Polish surveys. To further inspect the consistency of the femininity and masculinity scales, we plotted rating means (*M*s) and standard deviations (*SD*s). As expected, the observed distributions (see Fig 1) consistently pointed to a wide dispersion of the feminine and masculine word ratings around the scale midpoints, with a smaller dispersion at the scale endpoints, as well as a small dispersion of the neutral word ratings around the scale midpoints. There was also a high negative correlation between the femininity and masculinity ratings for the stereotypically feminine (*r* = –.85, *p* < .001), stereotypically masculine (*r* = –.75, *p* < .001), and neutral (*r* = –.51, *p* < .001) words (see Fig 2).

#### Word femininity and masculinity ratings

**Fig 3:**
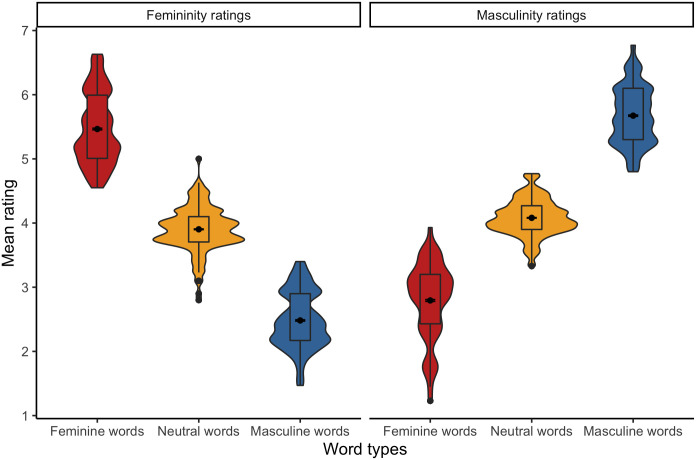
Mean femininity and masculinity ratings of Polish stereotypically feminine, neutral, and stereotypically masculine words.

**Fig 4:**
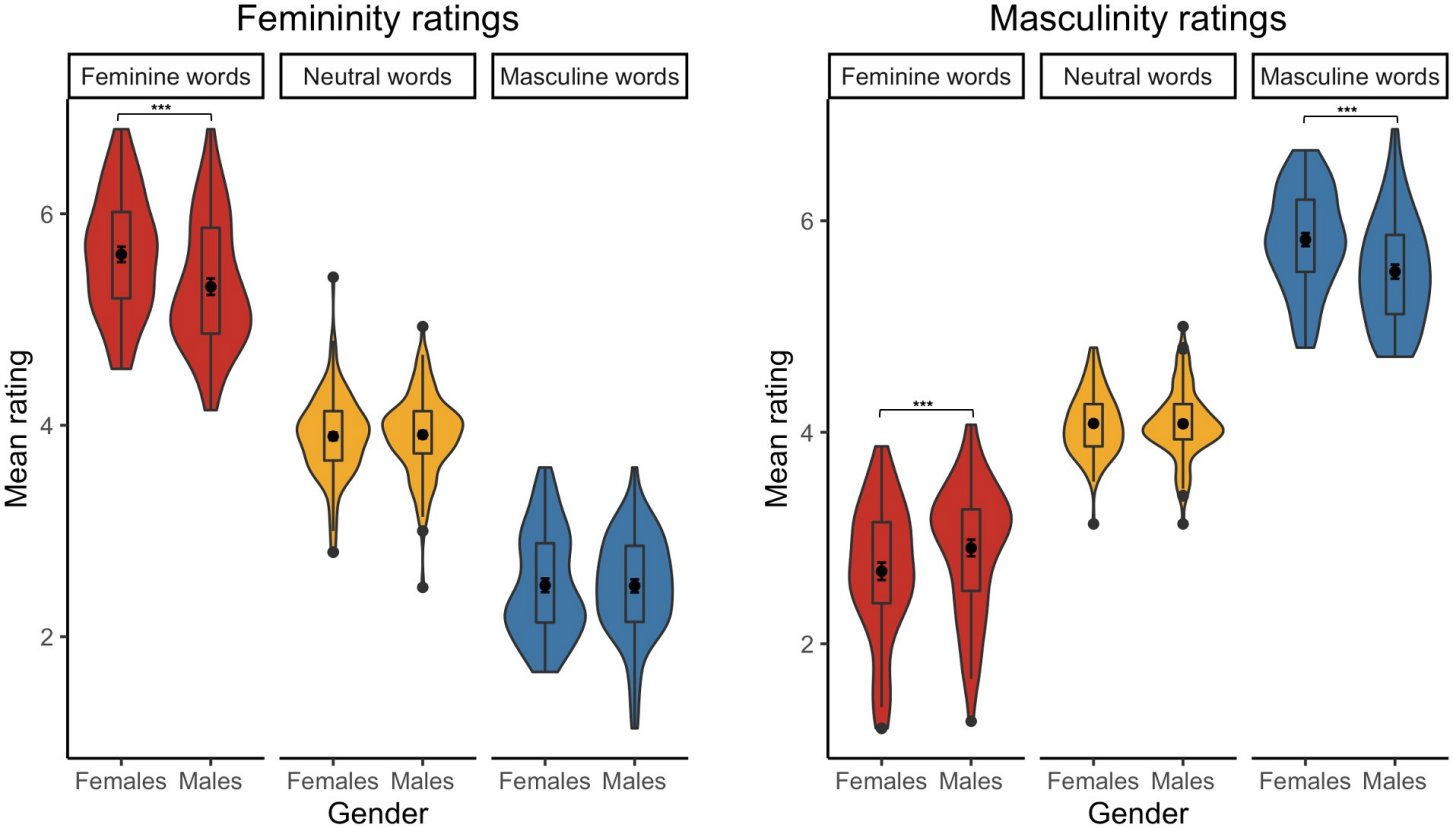
Mean femininity (left) and masculinity (right) ratings of Polish stereotypically feminine, neutral, and stereotypically masculine words from female and male participants [* p < .05, ** p < .01,*** p < .001].

The analysis performed on femininity ratings revealed a fixed effect of Word type, whereby stereotypically feminine words were rated as more feminine than both neutral words, *b* = 1.48, SE = .09, *t*(324.2) = 17.00, *p* < .001, and stereotypically masculine words, *b* = 2.84, SE = .13, *t*(277.4) = 21.17, *p* < .001. Then, stereotypically masculine words were rated as less feminine than neutral words, *b* = –1.36, SE = .09, *t*(325.3) = –15.38, *p* < .001 (see Fig 3). Moreover, the analysis also showed a Gender × Word type interaction, *b* = .31, SE = .12, *t*(144.6) = 2.69, *p* = .008. *Post-hoc* comparisons revealed that while there were no between-gender differences in the femininity ratings of neutral words, *b* = –.01, SE = .05, *t*(146.4) = –.24, *p* = .808, and stereotypically masculine words, *b* = –.04, SE = .12, *t*(145.4) = –.32, *p* = .747, stereotypically feminine words were rated as more feminine by females than by males, *b* = .30, SE = .12, *t*(144.7) = 2.57, *p* = .011 (see Fig 4). The reported effects for the Polish items were adjusted for word valence–a significant predictor of the femininity ratings, *b* = .11, SE = .03, *t*(236.19) = 4.15, *p* < .001. All means with 95% confidence intervals are provided in Table 2.

As for the masculinity ratings, the analysis also showed a fixed effect of Word type, whereby stereotypically masculine words were rated as more masculine than both neutral words, *b* = 1.61, SE = .07, *t*(236.0) = –24.35, *p* < .001, and stereotypically feminine words, *b* = –2.86, SE = .08, *t*(236.0) = –37.61, *p* < .001. Then, stereotypically feminine words were rated as less masculine than neutral words, *b* = –1.26, SE = .07, *t*(236.3) = –18.93, *p* < .001 (see Fig 3). The analysis also revealed a Gender × Word type interaction, *b* = –.52, SE = .28, *t*(142.20) = –3.00, *p* = .003; *b* = –.22, SE = .11, *t*(142.8) = –2.09, *p* = .039. *Post-hoc* comparisons showed that while there was no between-gender difference in the masculinity ratings of neutral words, *b* <–.01, SE = .05, *t*(223.4) = .08, *p* = .935, females rated stereotypically feminine words were rated as less masculine, *b* = –.22, SE = .06, *t*(424.6) = –3.95, *p* < .001, and stereotypically masculine words as more masculine, *b* = .31, SE = .06, *t*(424.2) = 5.57, *p* < .001, relative to males (see Fig 4). The reported effects for the Polish items were adjusted for word frequency–a significant predictor of the masculinity ratings, *b* = .14, SE = .04, *t*(232.4) = 3.71, *p* < .001. All means with 95% confidence intervals are provided in Table 3.

**Table 3:** Adjusted means (with 95% confidence intervals) of the femininity and masculinity ratings for the Polish stereotypically feminine, neutral, and stereotypically masculine words, specified by participants’ gender.

	Stereotypically feminine words (3 572 observations)	Stereotypically masculine words (3 578 observations)	Stereotypically neutral words (7 156 observations)
Mean femininity ratings	5.39 [5.23, 5.54]	2.55 [2.38, 2.71]	3.91 [3.82, 4.00]
*Females*	5.54 [5.34, 5.73]	2.53 [2.32, 2.73]	3.90 [3.80, 4.00]
*Males*	5.24 [5.05, 5.43]	2.57 [2.36, 2.77]	3.92 [3.81, 4.02]
Mean masculinity ratings	2.81 [2.70, 2.93]	5.68 [5.57, 5.79]	4.07 [3.99, 4.15]
*Females*	2.70 [2.58, 2.83]	5.83 [5.71, 5.95]	4.07 [3.98, 4.17]
*Males*	2.92 [2.80, 3.05]	5.52 [5.40, 5.65]	4.07 [3.97, 4.16]

#### The role of psycholinguistic variables in word ratings

The analyses were conducted separately for femininity and masculinity word ratings. As for the femininity ratings, the ordinal logistic regression revealed that, holding all other predictor variables constant, the odds of rating a stereotypically feminine item as feminine increased by 1.82 (95% CI [1.48, 2.23]) for a one-unit increase in arousal, by 1.37 (95% CI [1.03, 1.83]) for the items in a grammatical feminine relative to neutral form, as well as by 1.24 (95% CI [1.09, 1.42]) for a one-unit increase in valence. Moreover, these odds decreased by 0.64 (95% CI [.53,.78]) for a one-unit increase in concreteness, by 0.62 (95% CI [.52,.75]) for a one-unit increase in frequency, as well as by 0.61 (95% CI [.45,.82]) for the items in a grammatical masculine relative to neutral form. Second, the odds of rating a stereotypically masculine item as feminine increased by 1.32 (95% CI [1.24, 1.42]) for a one-unit increase in concreteness. Also, these odds decreased by 0.87 (95% CI [.80,.94]) for a one-unit increase in arousal as well as by 1.76 (95% CI [1.37, 2.26]) for the items in a grammatical plural relative to neutral form. Third, the odds of rating a neutral item as feminine increased by 1.25 (95% CI [1.16, 1.36]) for a one-unit increase in valence, by 1.31 (95% CI [1.17, 1.47]) for a one-unit increase in concreteness, by 1.20 (95% CI [1.07, 1.35]) for a one-unit increase in the number of syllables as well as by 1.45 (95% CI [1.15, 1.86]) for the items in a grammatical feminine relative to neutral form. Furthermore, these odds decreased by 0.84 (95% CI [.73,.97]) for a one-unit increase in arousal, by 0.79 (95% CI [.74,.84]) for a one-unit increase in age of acquisition as well as by 0.75 (95% CI [.53,.88]) for the items in a grammatical masculine compared to neutral form. The estimates for all significant predictors are provided in Table 4.

**Table 4:** The results of ordinal regression for the Polish items, showing the associations between the femininity ratings and the psycholinguistic variables.

	Stereotypically feminine words			Stereotypically masculine words			Stereotypically neutral words		
	*b*	95% CI	*z*	*b*	95% CI	*z*	*b*	95% CI	*z*
Frequency	–.56	[–.65,–.47]	***–12.48	.57	[.49,.67]	***12.66	–	–	–
Valence	.55	[.50,.60]	***20.92	–.37	[–.42.–.32]	***–14.15	.23	[.15,.31]	***5.52
Arousal	–.14	[–.22,–.06]	**–3.47	.30	[.22,.38]	***7.26	–.18	[–.32,–.03]	*–2.43
Concreteness	.28	[.21,.35]	***7.95	–.30	[–.37,–.23]	***–8.27	.27	[.15,.39]	***4.60
Age of acquisition	–.25	[–.29,–.21]	***–12.54	.26	[.23,.30]	***13.18	–	[–.30,–.17]	***–7.08
Syllables	.08	[.00,.14]	*2.21	–	–	–	.18	[.06,.30]	**3.03
Letters	–	–	–	–.07	[–.10,–.04]	***–4.97	–	–	–
Grammatical form^1^:									
*Fem vs*. *Neu*	.18	[.04,.33]	*2.50	–	–	–	.38	[.14,.62]	**3.06
*Masc vs*.*Neu*	–.29	[–.45,–.14]	***–3.75	.22	[.07,.36]	**2.89	–.38	[–.64,–.13]	**–2.92
*Pl vs*. *Neu*	.56	[.31,.82]	***4.40	–.45	[–.70,–.20]	***–3.48	–	–	–

* *p* < .05,

** *p* < .01,

*** *p* < .001.

Note.

^1^Fem = feminine, Masc = masculine, Neu = neutral, Pl = plural

**Fig 5:**
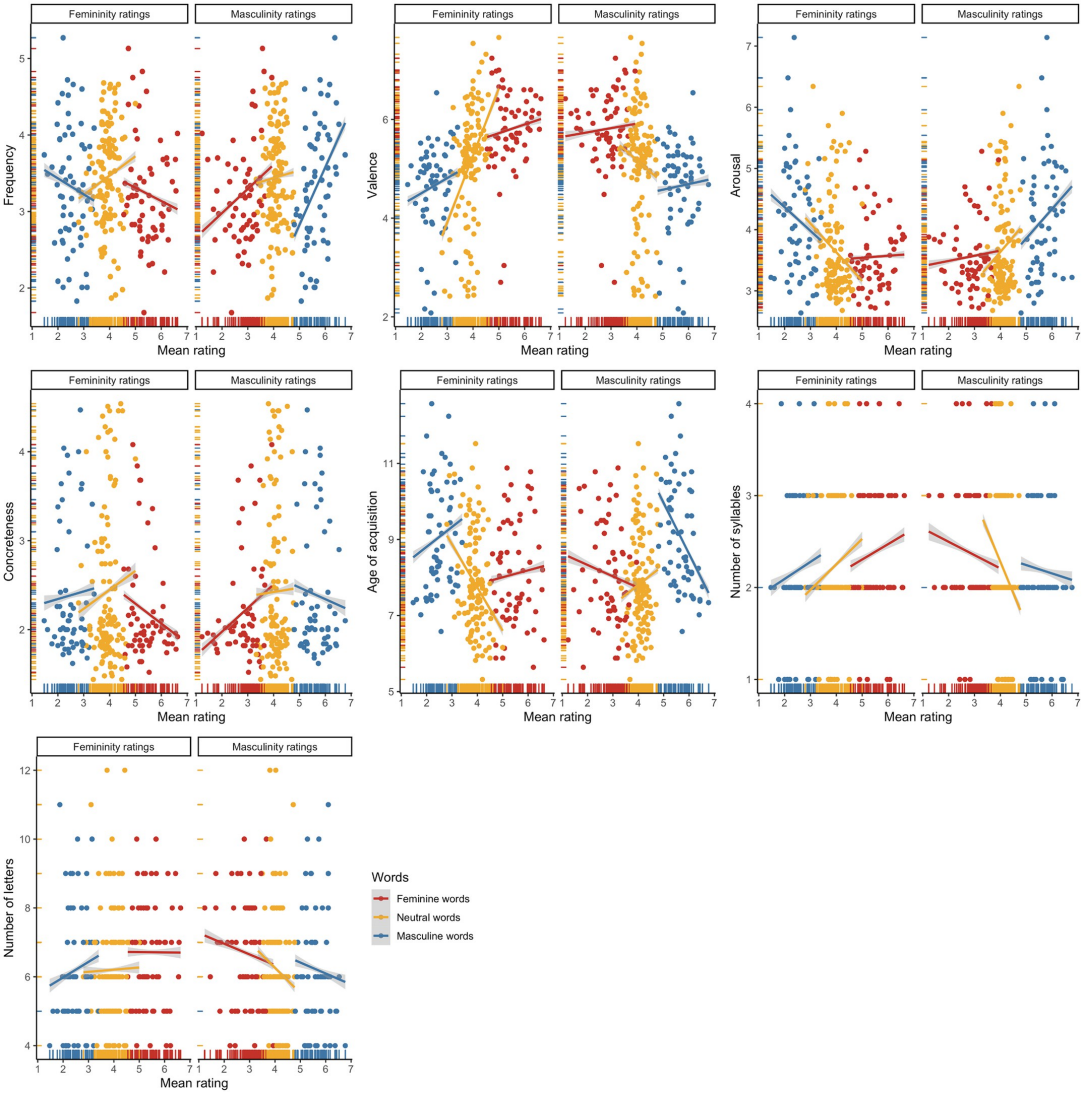
Associations among the mean femininity and masculinity ratings of the Polish items and the controlled psycholinguistic variables.

As for the masculinity ratings, the ordinal logistic regression for the masculinity ratings revealed that, holding all other predictor variables constant, the odds of rating a stereotypically feminine item as masculine increased by 1.51 (95% CI [1.30, 1.74]) for a one-unit increase in frequency, by 1.62 (95% CI [1.33, 1.96]) for a one-unit increase in concreteness, by 0.71 (95% CI [0.53, 0.94]) for the items in a grammatical feminine relative to neutral form as well as by 1.35 (95% CI [1.01, 1.81]) for the items in a grammatical masculine relative to neutral form. Moreover, these odds decreased by 0.64 (95% CI [.52,.78]) for a one-unit increase in arousal. Second, the odds of rating a stereotypically masculine item as masculine increased by 1.33 (95% CI [1.15, 1.54]) for a one-unit increase in valence, by 1.81 (95% CI [1.55, 2.13]) for a one-unit increase in arousal as well as by 14.51 (95% CI [6.04, 39.04]) for the items in a grammatical plural relative to neutral form. Also, these odds decreased by 0.74 (95% CI [.63,, 87]) for a one-unit increase in concreteness and by 0.87 (95% CI [.81,.93]) for a one-unit increase in age of acquisition. Third, the odds of rating a neutral item as feminine increased by 1.28 (95% CI [1.13, 1.45]) for a one-unit increase in arousal as well as by 1.13 (95% CI [1.06, 1.21]) for a one-unit increase in age of acquisition. Furthermore, these odds decreased by 0.88 (95% CI [0.79,.99]) for a one-unit increase in concreteness, by 0.69 (95% CI [.61,.78]) for a one-unit increase in the number of syllables as well as by 0.66 (95% CI [.52,.85]) for the items in a grammatical feminine relative to neutral form. The estimates for all significant predictors are provided in Table 5. Moreover, the results of the correlational analyses for the Polish items are provided in Table 6 and Fig 5.

**Table 5:** The results of ordinal regression for the Polish items, showing the associations between the masculinity ratings and the psycholinguistic variables.

	Stereotypically feminine words			Stereotypically masculine words			Stereotypically neutral words		
	*b*	95% CI	*z*	*b*	95% CI	*z*	*b*	95% CI	*z*
Frequency	.41	[.26,.56]	***–5.47	–	–	–	–	–	–
Valence	–	–	–	.28	[.14,.43]	***7.40	–	–	–
Arousal	–.45	[–.65,–.24]	***–4.31	.59	[.44,.75]	***–3.69	.25	[.12,.37]	***3.83
Concreteness	.48	[.29,.67]	***4.94	–.30	[–.47,–.13]	***–3.69	–.12	[–.23,–.01]	*–2.19
Age of acquisition	–	–	–	–.14	[–.21,–.07]	***–4.02	.13	[–.30,–.19]	***3.77
Syllables	–	–	–	–	–	–	–.37	[–.49,–.24]	***–5.88
Letters	–	–	–	–	–	–	–	–	–
Grammatical form^1^:									
*Fem vs*. *Neu*	.30	[–.63,–.06]	*–2.39	–	–	–	–.41	[–.66,–.16]	**–3.22
*Masc vs*.*Neu*	.37	[.01,.60]	*2.04	–	–	–	–	–	–
*Pl vs*. *Neu*	–	–	–	–2.67	[1.80, 3.66]	***5.69	–	–	–

* *p* < .05,

** *p* < .01,

*** *p* < .001.

Note.

^1^Fem = feminine, Masc = masculine, Neu = neutral, Pl = plural

**Table 6:** Pearson correlation coefficients (*r*) showing the associations among the femininity and masculinity scores and the psycholinguistic variables for the Polish feminine, masculine, and neutral words.

Stereotypically feminine words	1	2	3	4	5	6	7	8
1 Femininity score								
2 Masculinity score	***–.85							
3 Frequency	–	*.28						
4 Valence	–	–	***.49					
5 Arousal	–	–	**.43	–				
6 Concreteness	–	*.27	–	–	***.58			
7 Age of acquisition	–	–	**–.46	**–.36	–	–		
8 Syllables	–	–	*–.29	–	–	–	–	
9 Letters	–	–	**–.38	–	–	–	–	***.74
**Stereotypically masculine words**								
1 Femininity score								
2 Masculinity score	***–.75							
3 Frequency	–	**.43						
4 Valence	–	–	–					
5 Arousal	–	–	**.42	***–.70				
6 Concreteness	–	–	–	***–.58	***.66			
7 Age of acquisition	–	**–.43	***–.56	*–.27	–	–		
8 Syllables	–	–	*–.32	–	–	–	–	
9 Letters	–	–	*–.32	–	–	–	–	***.84
**Stereotypically neutral words**								
1 Femininity score								
2 Masculinity score	***–.51							
3 Frequency	–	–						
4 Valence	***.41	–	**.33					
5 Arousal	*–.21	*.18	–	***–.47				
6 Concreteness	–	–	***–.40	–	***.56			
7 Age of acquisition	**–.32	–	**–.15	*–.28	–	*.27		
8 Syllables	–	**–.26	–	–	–	–	*.18	
9 Letters	–	–	–	–	*.20	***.35	*.20	***.73

* *p* < .05,

** *p* < .01,

*** *p* < .001

#### The role of gender role identities in word ratings

The analysis of femininity ratings revealed a fixed effect of Word type, whereby stereotypically feminine words were rated as more feminine than both neutral words, *b* = –1.53, SE = .09, *t*(322.6) = –17.49, *p* < .001, and stereotypically masculine words, *b* = 2.91, SE = .13, *t*(269.7) = 22.42, *p* < .001. Then, stereotypically masculine words were rated as less feminine than neutral words, *b* = 1.37, SE = .09, *t*(326.8) = 15.66, *p* < .001 (see Fig 3 and Table 3).

**Fig 6:**
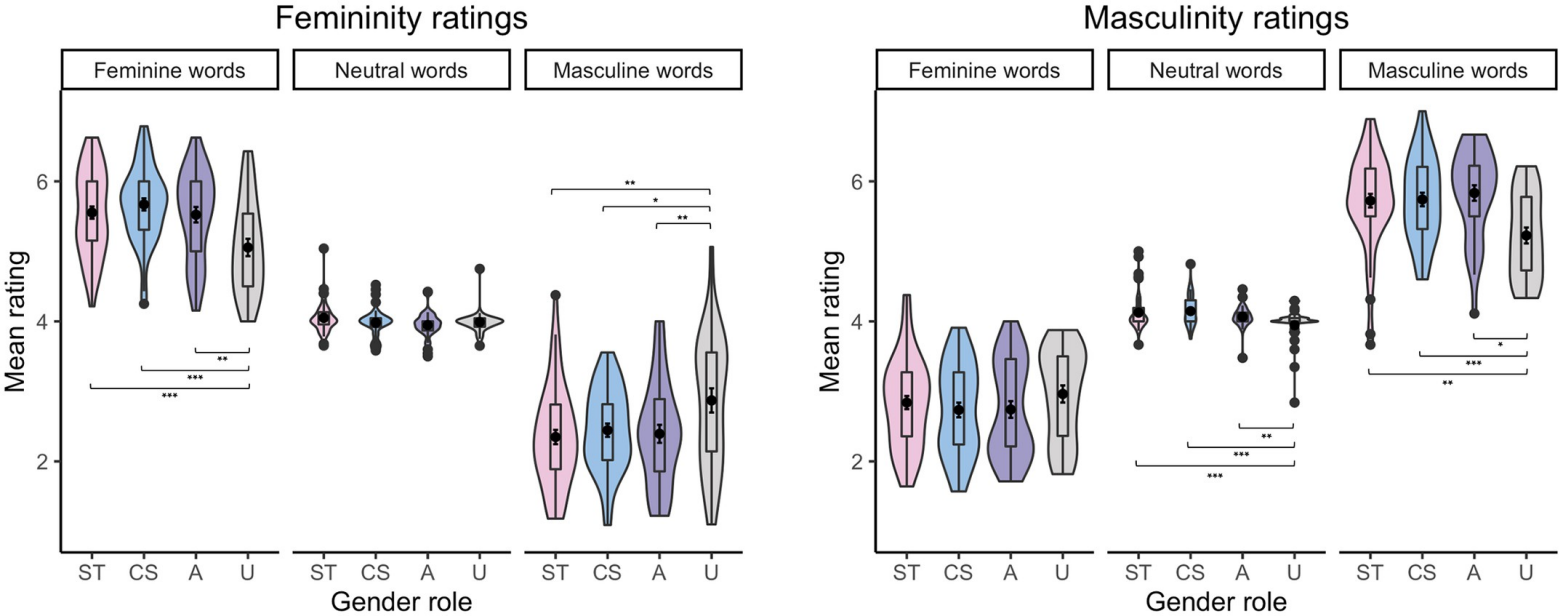
Mean femininity (left) and masculinity (right) ratings of Polish stereotypically feminine, neutral, and masculine words from participants with the sex-typed (ST), cross-sex (CS), androgynous (A), and undifferentiated (U) gender role identities [* p < .05, ** p < .01,*** p < .001].

The analysis of femininity ratings also showed a Word type × Gender role interaction. Regarding stereotypically feminine words, *post-hoc* comparisons revealed that undifferentiated individuals rated them as less feminine than sex-typed individuals, *b* = –.57, SE = .16, *t*(144.9) = –3.59, *p <* .001, than cross-sex individuals, *b* = –.71, SE = .17, *t*(142.5) = –4.21, *p <* .001, and than androgynous individuals, *b* = –.58, SE = .17, *t*(144.6) = –3.30, *p =* .001 (see Fig 6 and Table 7). Regarding stereotypically masculine words, *post-hoc* comparisons demonstrated that undifferentiated individuals rated them as less feminine than sex-typed individuals, *b* = .55, SE = .17, *t*(145.9) = 3.20, *p =* .002, than cross-sex individuals, *b* = .44, SE = .18, *t*(144.3) = 2.40, *p =* .018, and than androgynous individuals, *b* = .50, SE = .19, *t*(144.9) = 2.66, *p =* .009 (see Fig 6 and Table 7).

**Table 7:** Adjusted means (with 95% confidence intervals) of the femininity and masculinity ratings for the Polish stereotypically feminine, neutral, and stereotypically masculine words, specified by participants’ gender role identities.

	Stereotypically feminine words	Stereotypically neutral words	Stereotypically masculine words
Mean femininity ratings			
*Sex-typed*	5.54 [5.32, 5.76]	3.94 [3.82, 4.06]	2.33 [2.10, 2.56]
*Cross-sex*	5.67 [5.43, 5.91]	3.93 [3.80, 4.05]	2.45 [2.20, 2.71]
*Androgynous*	5.54 [5.28, 5.79]	3.91 [3.78, 4.05]	2.39 [2.12, 2.66]
*Undifferentiated*	4.96 [4.69, 5.23]	3.80 [3.66, 3.94]	2.89 [2.60, 3.18]
Mean masculinity ratings			
*Sex-typed*	2.84 [2.63, 3.05]	4.12 [4.03, 4.22]	5.74 [5.54, 5.94]
*Cross-sex*	2.73 [2.50, 2.96]	4.14 [4.04, 4.25]	5.73 [5.51, 5.95]
*Androgynous*	2.72 [2.47, 2.96]	4.07 [3.97, 4.18]	5.85 [5.62, 6.09]
*Undifferentiated*	2.94 [2.69, 3.20]	3.94 [3.83, 4.05]	5.23 [4.98, 5.48]

The analysis of masculinity ratings revealed a fixed effect of Word type, whereby stereotypically masculine words were rated as more masculine than both neutral words, *b* = –1.57, SE = .08, *t*(319.6) = –19.63, *p* < .001, and stereotypically feminine words, *b* = –2.82, SE = .12, *t*(289.7) = –24.40, *p* < .001. Then, stereotypically feminine words were rated as less masculine than neutral words, *b* = 1.26, SE = .08, *t*(322.7) = 15.21, *p* < .001 (see Fig 3 and Table 3).

The analysis of masculinity ratings also showed a fixed effect of Gender role, such that undifferentiated individuals rated words as less masculine than sex-typed individuals, *b* = –.19, SE = .06, *t*(146.5) = –3.02, *p =* .003, than cross-sex individuals, *b* = –.16, SE = .07, *t*(144.2) = –2.44, *p =* .016, and than androgynous individuals, *b* = –.17, SE = .07, *t*(145.7) = –2.52, *p =* .013.

The analysis of masculinity ratings also revealed a Word type × Gender role interaction. Regarding stereotypically neutral words, *post-hoc* comparisons indicated that undifferentiated individuals rated them as less masculine than sex-typed individuals, *b* = –.18, SE = .06, *t*(145.0) = –3.29, *p <* .001, cross-sex individuals, *b* = –.20, SE = .06, *t*(144.7) = –3.48, *p <* .001, and androgynous individuals, *b* = –.13, SE = .06, *t*(143.3) = –2.21, *p =* .029 (see Fig 6 and Table 6). Regarding stereotypically masculine words, *post-hoc* comparisons also indicated that undifferentiated individuals rated them as less masculine than sex-typed individuals, *b* = –.51, SE = .15, *t*(142.4) = –3.47, *p <* .001, than cross-sex individuals, *b* = –.50, SE = .15, *t*(140.6) = –3.25, *p =* .001, and than androgynous individuals, *b* = –.62, SE = .16, *t*(141.3) = –3.90, *p <* .001 (see Fig 6 and Table 7).

#### The role of nouns’ grammatical gender in word ratings

First, the analysis of femininity ratings showed a fixed effect of Word type, whereby stereotypically feminine relative to masculine words were rated as more feminine, *b* = 2.88, *t*(219.5) = 20.46, *p* < .001. Then, there was a fixed effect of Grammatical gender, such that grammatically feminine relative to masculine words were rated as more feminine, *b* = .30, *t*(109.9) = 3.10, *p* = .003. There was also a Word type × Grammatical gender interaction, *b* = .61, *t*(107.0) = 3.13, *p* = .002. *Post-hoc t*-tests revealed that grammatically feminine compared to masculine words were rated as more feminine when they were stereotypically feminine, *b* = .61, *t*(108.0) = 4.20, *p* < .001, with no difference for stereotypically masculine words, *b* <–.01, *t*(108.7) <–.01, *p* = .999.

**Fig 7:**
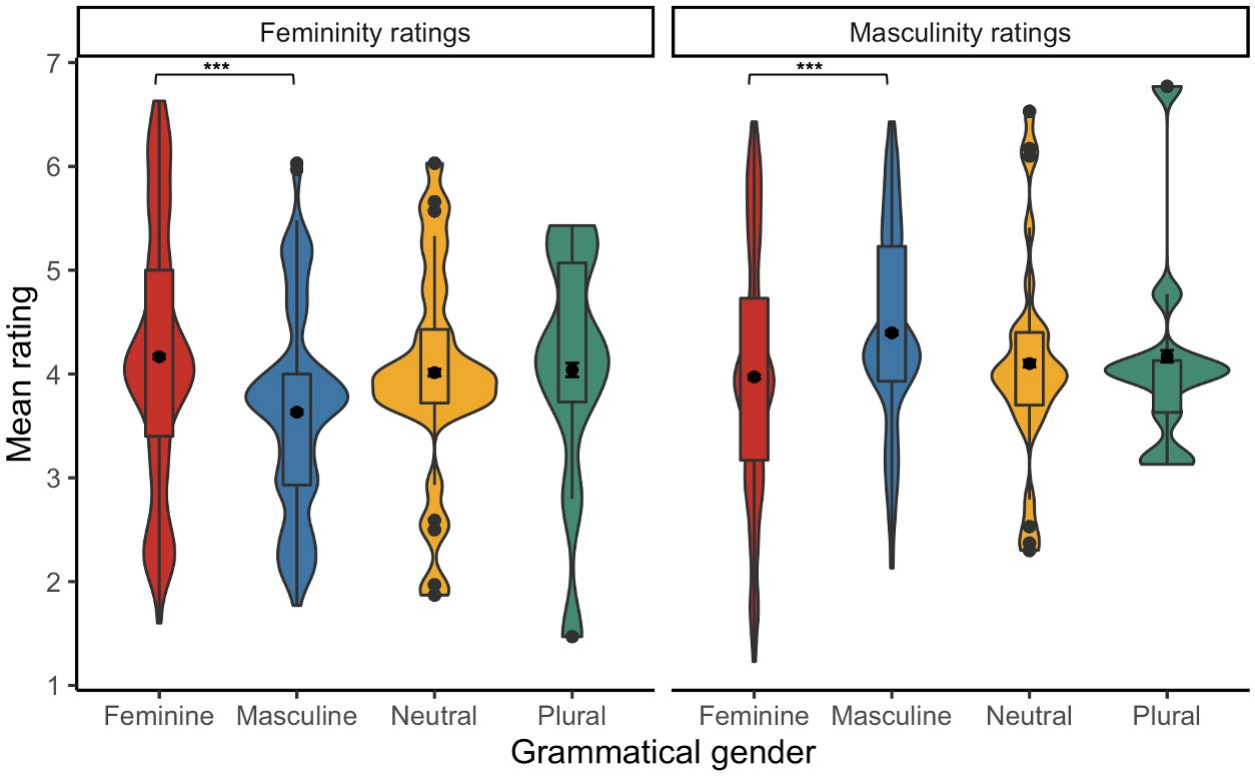
Mean femininity (left) and masculinity (right) ratings for grammatically feminine, masculine, neutral, and plural Polish items [* p < .05, ** p < .01,*** p < .001].

Then, the analysis of masculinity ratings showed a fixed effect of Word type, whereby stereotypically masculine relative to feminine words were rated as more masculine, *b* = –2.81, *t*(205.4) = –21.46, *p* < .001. Then, there was a fixed effect of Grammatical gender, such that grammatically masculine relative to feminine words were rated as more masculine, *b* = –.21, *t*(103.7) = –2.10, *p* = .038. There was also a Word type × Grammatical gender interaction, *b* = –.59, *t*(103.4) = –2.99, *p* = .004. *Post-hoc t*-tests showed that grammatically masculine compared to feminine words were rated as more masculine when they were stereotypically feminine, *b* = –.51, *t*(103.9) = –3.44, *p* < .001, with no difference for stereotypically masculine words, *b* = .13, *t*(103.9) = .65, *p* = .514 (see Fig 7 and Table 8).

**Table 8:** Mean femininity and masculinity ratings (with 95% confidence intervals) for Polish stereotypically feminine and stereotypically masculine words, specified by grammatical gender.

	Masculinity ratings		Femininity ratings	
	Grammatically feminine words	Grammatically masculine words	Grammatically feminine words	Grammatically masculine words
Stereotypically feminine words	2.57 [2.38, 2.77]	3.08 [2.83, 3.33]	5.70 [5.50, 5.89]	5.09 [4.84, 5.34]
Stereotypically masculine words	5.68 [5.46, 5.89]	5.59 [5.39, 5.79]	2.51 [2.29, 2.73]	2.51 [2.30, 2.72]

## Discussion

The present survey research on Polish nouns was aimed to test the degree of gender stereotype load ingrained in single words that were pre-selected as stereotypically associated with either males or females. To this end, participants assessed stereotypically feminine, stereotypically masculine, and stereotypically neutral (control) words on the femininity and masculinity scales. Due to the fact that Polish belongs to the group of grammatical gender languages, whereby lexical items are marked for gender, we expected a high degree of stereotype load that would be evident when exposed to nouns that only stereotypically, yet not definitionally, denote females or males.

In agreement with our expectations, Polish stereotypical gender nouns were assessed in line with the categories ascribed to them. Namely, while stereotypically feminine words were rated as more feminine than stereotypically masculine items on the femininity scale, the reversed pattern was observed on a masculinity scale, whereby stereotypically masculine nouns were evaluated as more masculine compared to stereotypically feminine words. At the same time, stereotypically neutral (control) nouns were consistently perceived as neither feminine nor masculine. First of all, such results confirm that the single nouns included in our database of stereotypically feminine, stereotypically masculine, and stereotypically neutral words represent lexical items that are not only well-matched on a number of psycholinguistic variables (see Table 2) but are also well-selected to illustrate the degree of gender stereotypes assigned to single words. Secondly, the results strongly indicate that exposure to a single lexical item that does not definitionally denote a woman or a man is enough to activate gender stereotypes, therefore reflecting a high degree of stereotype load.

Importantly, even though the general effect described above was independent of a grammatical gender, we ran exploratory analyses with grammatical gender as a factor in order to address the potential role of Polish grammatical gender in the stereotype load of the items tested. As expected, the results revealed that stereotypically feminine nouns of a feminine grammatical gender were rated higher on a femininity scale relative to those of a masculine, neutral, or plural grammatical gender. Similarly, stereotypically masculine nouns of a masculine grammatical gender elicited higher ratings on a masculinity scale. Such results therefore point to a potentially bi-directional role of a grammatical gender, which not only reflects gender stereotypes but also reinforces them.

Interestingly, the results also yielded an effect driven by participants’ gender, whereby females assessed stereotypically feminine and stereotypically masculine nouns higher on femininity and masculinity scales, respectively. In contrast, male participants were somewhat more neutral in their assessments. Such results suggest that for females the stereotypical load is more pronounced than for males, which is consistent with previous research findings indicating that females perceive gender differences as greater than males (e.g., [42, 43]).

Furthermore, the analyses performed on the BSRI [57] results revealed an effect of participants’ gender roles, whereby individuals with an undifferentiated gender role (i.e., low on both feminine and masculine gender role scale) were more neutral in their assessment of the stereotypicality level relative to those occupying sex-typed, cross-sex typed, and androgynous gender roles. This indicates that participants with an undifferentiated gender role might be less sensitive to gender stereotypes [50].

The aim of Study II is to further test whether and to what extent the effects observed for Polish (a grammatical gender language) are reflected in English (i.e., a natural gender language).

## Study II: The perception of English nouns

### Method

#### Participants

In total, 199 native speakers of English participated in Study II, 30 of whom were excluded from the analyses due to providing incomplete answers or following a response set (see Study I). This resulted in the final sample of 169 participants (88 females, 81 males) between the ages of 18 and 40 (*M* = 26.60; 95% CI [25.81, 27.40]). About 33% of the participants had completed high school as their highest education level, of whom the majority were continuing education at a university level. Nearly 50% of the sample had completed undergraduate studies, whereas over 10% were holders of a Master’s degree. In terms of their gender role identities, participants were classified as sex-typed (*n* = 44), cross-sex (*n* = 35), androgynous (*n* = 40), or undifferentiated (*n* = 50) individuals based on the BSRI [57].

#### Stimuli

Stimuli selection for Study II was performed in line with Study I, and was based on the SUBTLEX-UK database [71]. Table 2 provides examples of the stimuli used. Table 9 presents ranges, means, and confidence intervals for the psycholinguistic variables for which the target words were controlled and matched across conditions. In order to meet the selection criteria, the final stimuli list included 14.58% of Polish–English translation equivalents. The selected nouns were divided into six 90-item lists (30 words per condition) for the purposes of data collection.

**Table 9:** Ranges and means (with 95% confidence intervals) for the psycholinguistic variables across the three word types for the English items.

	Range	Mean	Stereotypically feminine nouns (*n* = 60)	Stereotypically masculine nouns (*n* = 60)	Stereotypically neutral nouns (*n* = 120)
Frequency^1^	1.17–5.68	3.54 [3.46, 3.62]	3.43 [3.24, 3.62]	3.52 [3.35, 3.69]	3.61 [3.52, 3.70]
Valence^2^	1.30–8.05	5.32 [5.16, 5.48]	6.02 [5.84, 6.22]	4.79 [4.45, 5.13]	5.23 [5.00, 5.46]
Arousal^2^	1.75–7.24	3.95 [3.83, 4.06]	3.85 [3.66, 4.05]	4.11 [3.88, 4.34]	3.91 [3.74, 4.07]
Concreteness^3^	1.00–6.60	2.01 [1.85, 2.16]	1.79 [1.59, 1.99]	1.95 [1.77, 2.13]	2.15 [1.88, 2.42]
Age of acquisition^4^	2.50–14.21	7.21 [6.93, 7.49]	6.81 [6.31, 7.30]	7.94 [7.41, 8.46]	7.04 [6.63, 7.45]
Letters	5–12	7.40 [7.20, 7.61]	7.57 [7.16, 7.98]	7.10 [6.74, 7.46]	7.48 [7.18, 7.79]
Syllables	1–4	2.26 [2.37, 2.55]	2.37 [2.18, 2.55]	2.23 [2.09, 2.38]	2.22 [2.06, 2.37]

Scales and databases:

^1^Frequency: 1 (*low*)– 7 (*high*) scale, SUBTLEX-EN [71].

^2^Valence and Arousal: 1 (*negative/unarousing*)– 9 (*positive/arousing*) scales [60].

^3^Concreteness: 1 (*concrete*)– 9 (*abstract*) scale [61] (the original 1–5 scale reversed and transformed to the 1–9 scale).

^4^Age of Acquisition [72].

#### Procedure

The procedure was the same as in Study I, with the exception of language (Polish in Study I, English in Study II).

#### Data analysis

Data analyses for English items were performed in line with those run for Polish words (see section 2.1.4. for details).

### Results

#### Reliability of the ratings

**Fig 8:**
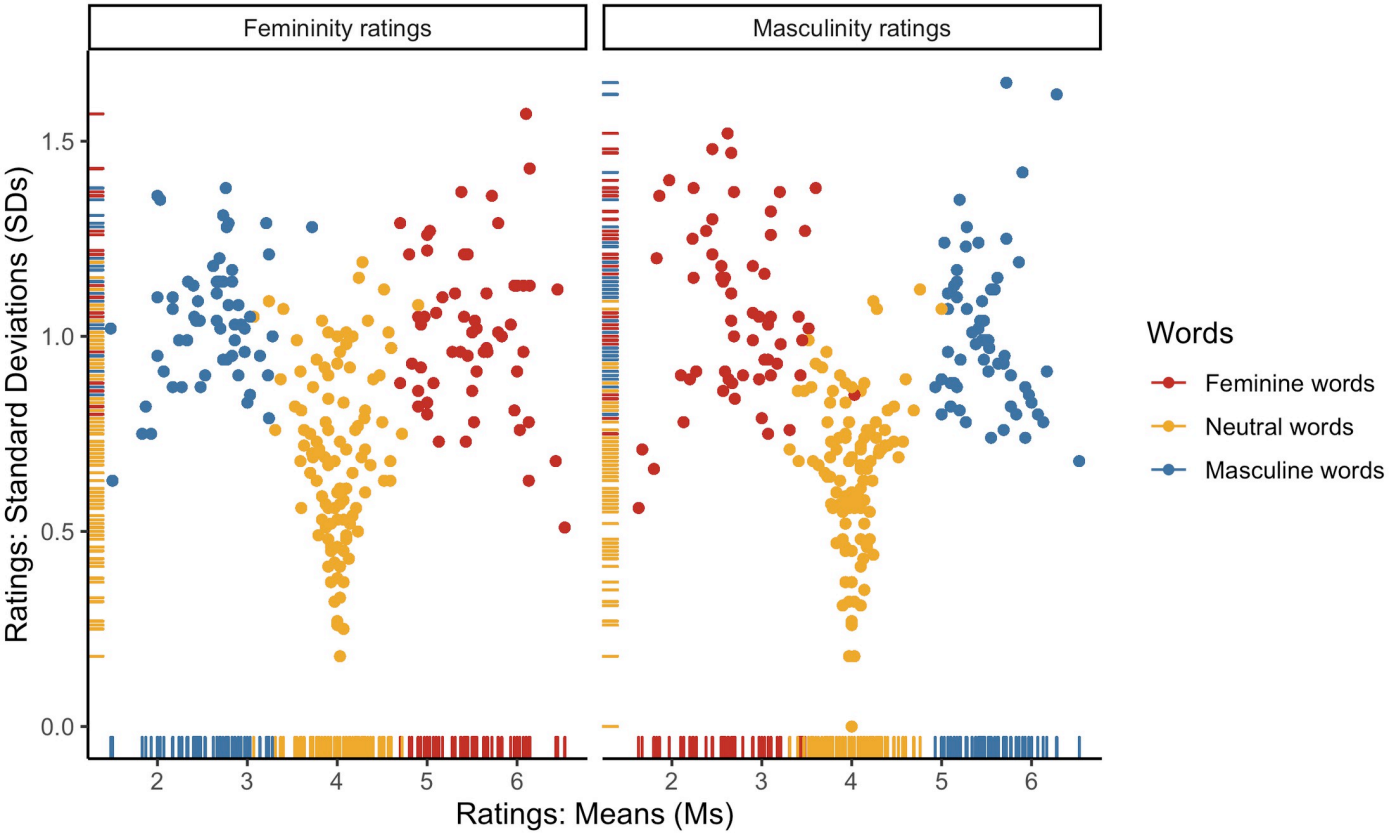
Distributions of rating means (Ms) and standard deviations (SDs) for the English items: (i) a left-biased distribution of the femininity ratings of the stereotypically masculine words and the masculinity ratings of the stereotypically feminine words; (ii) a right-biased distribution of the femininity ratings of the stereotypically feminine words and the masculinity ratings of the stereotypically masculine words; (iii) a center-biased distribution of the femininity and masculinity ratings of the neutral words.

**Fig 9:**
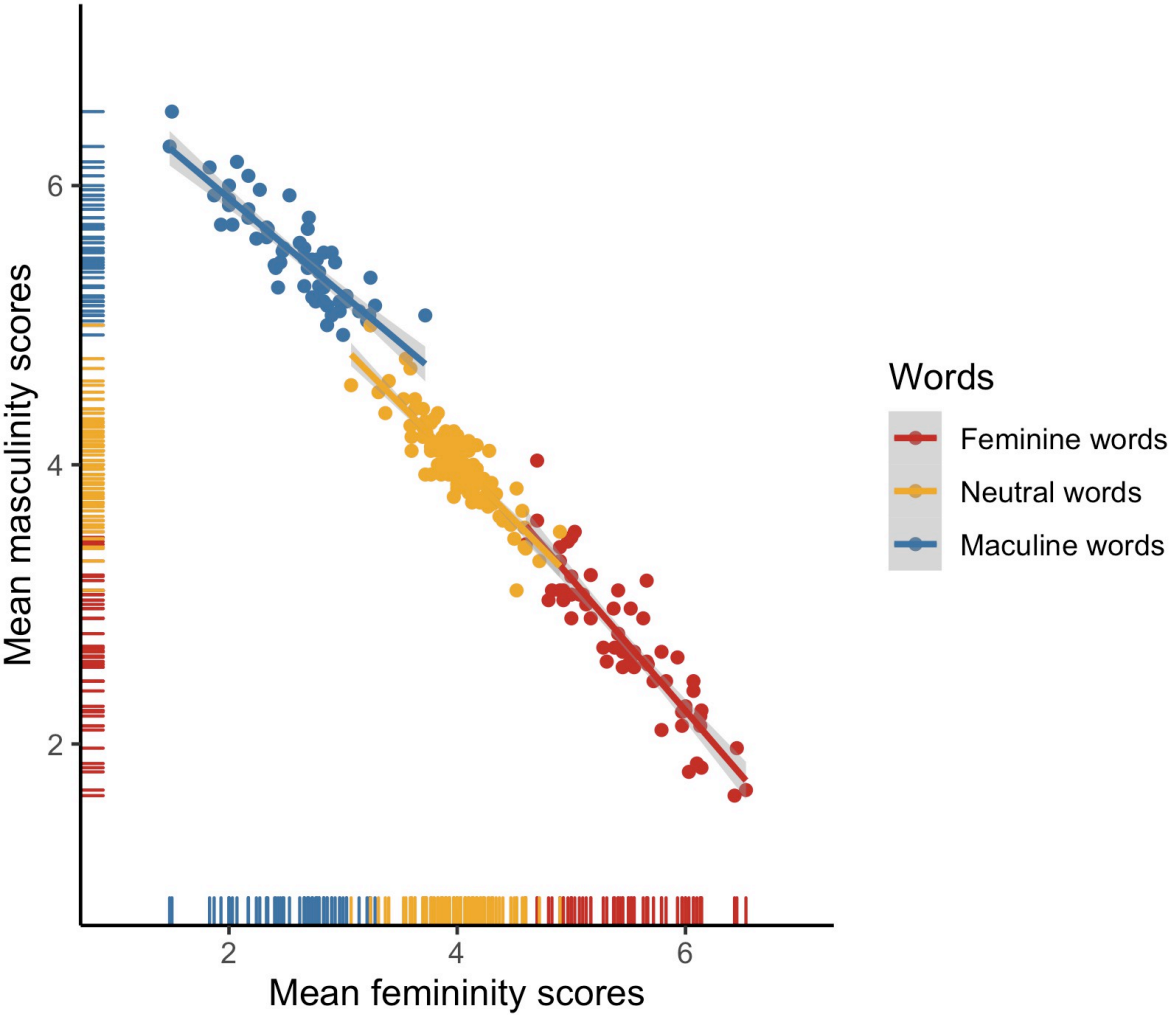
Associations between the mean masculinity and femininity scores for the English items.

ICCs pointed to high inter-rater reliability for both the femininity (ICC = .94) and masculinity ratings of English items (ICC = .93). Furthermore, both the femininity (*α =* .72) and masculinity (*α =* .74) scales demonstrated adequate degrees of internal consistency across all English surveys. To further inspect the consistency of the femininity and masculinity scales, we plotted rating means (*M*s) and standard deviations (*SD*s). As expected, the observed distributions (see Fig 8) consistently pointed to a wide dispersion of the feminine and masculine word ratings around the scale midpoints, with a smaller dispersion at the scale endpoints, as well as a small dispersion of the neutral word ratings around the scale midpoints. There was also a high negative correlation between the femininity and masculinity ratings for the stereotypically feminine (*r* = –.91, *p* < .001), stereotypically masculine (*r* = –.87, *p* < .001), and neutral (*r* = –.87, *p* < .001) words (see Fig 9).

#### Femininity and masculinity ratings

**Fig 10:**
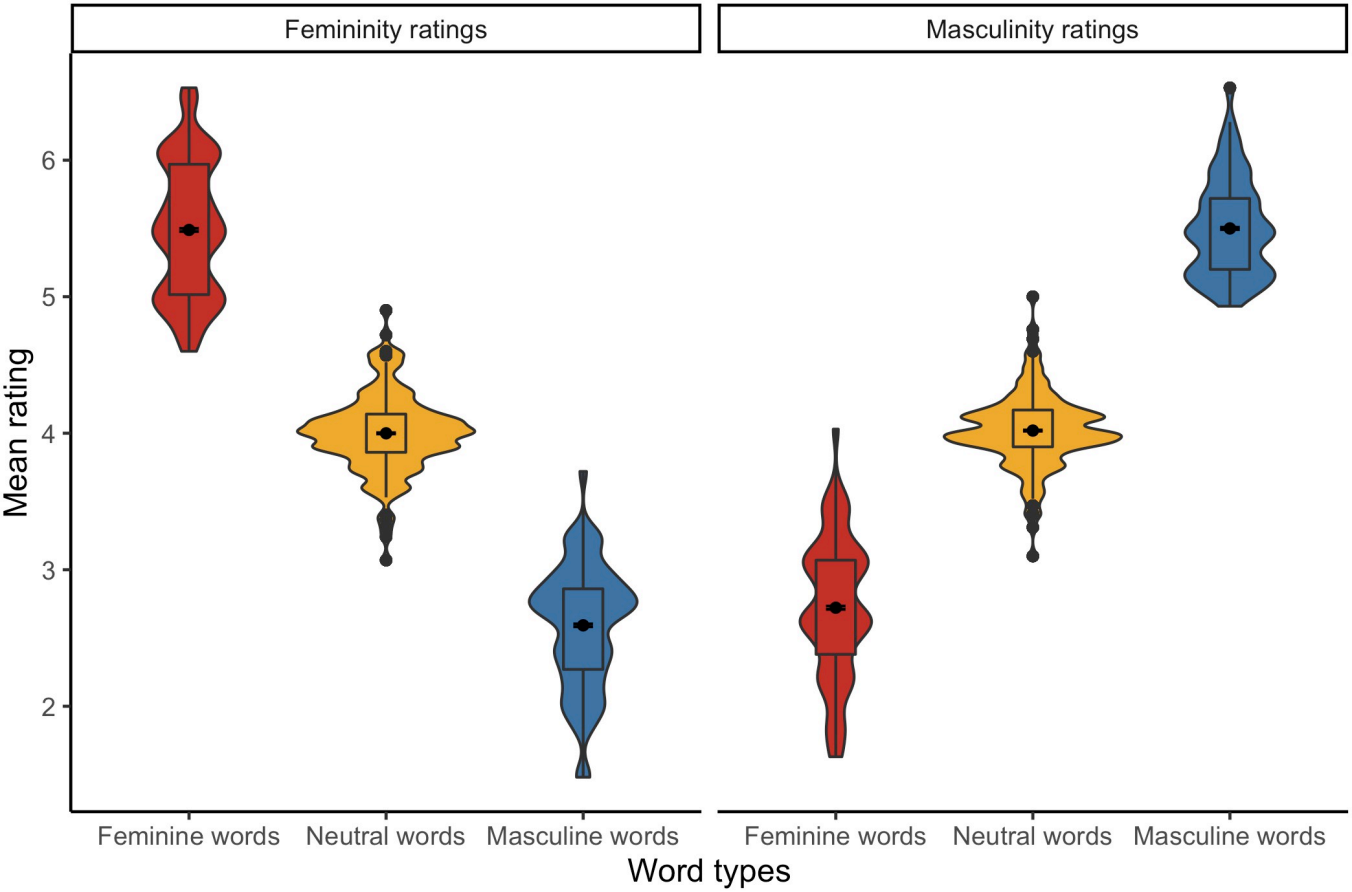
Mean femininity and masculinity ratings of English stereotypically feminine, neutral, and stereotypically masculine words.

**Fig 11:**
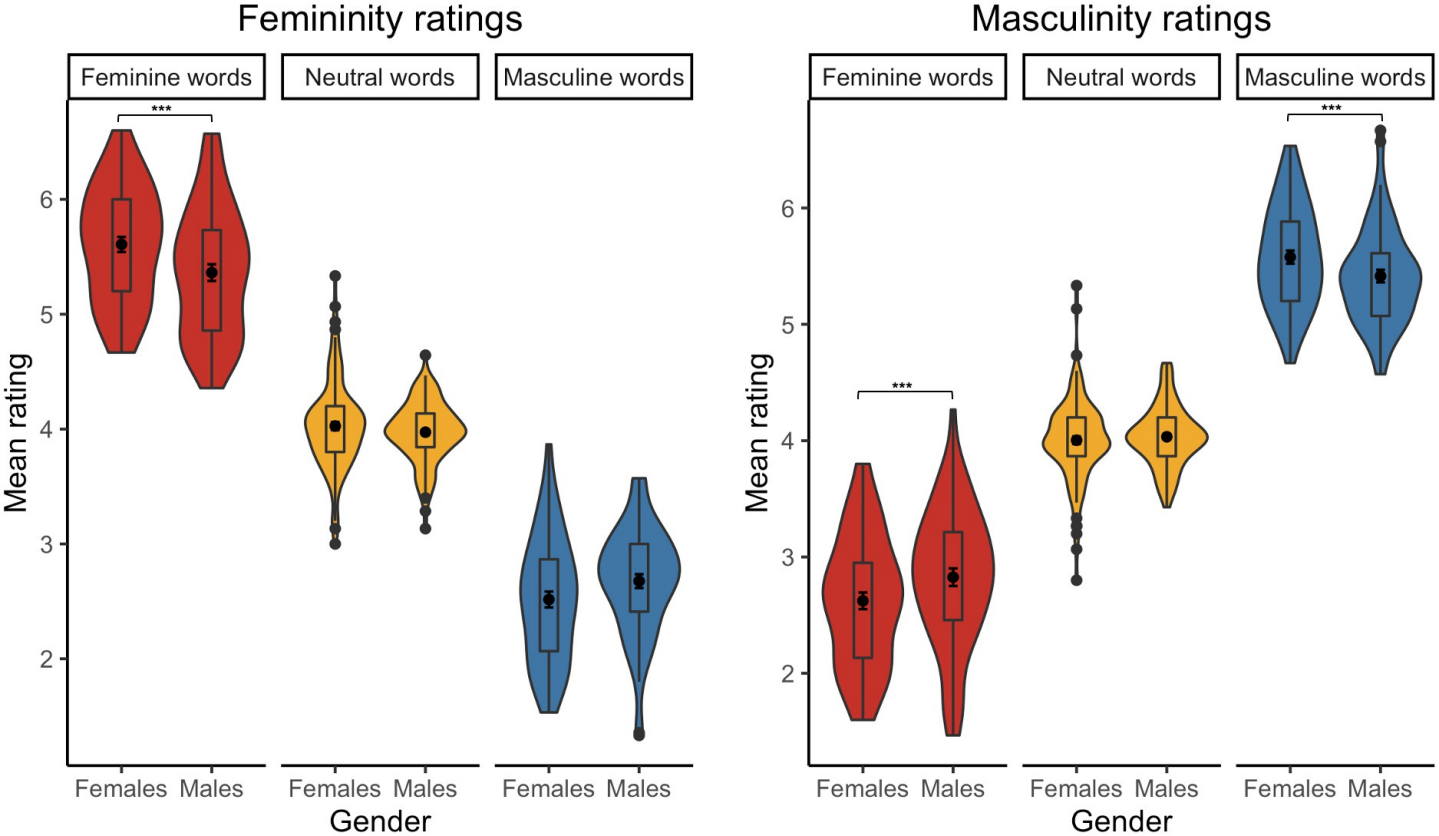
Mean femininity (left) and masculinity (right) ratings of English stereotypically feminine, neutral, and stereotypically masculine words from female and male participants [* p < .05, ** p < .01, *** p < .001].

Regarding the femininity ratings of the English items, the analysis revealed a fixed effect of Word type, whereby stereotypically feminine words were rated as more feminine than both neutral words, *b* = 1.41, SE = .08, *t*(322.4) = 17.44, *p* < .001, and stereotypically masculine words, *b* = 2.77, SE = .12, *t*(260.1) = 22.53, *p* < .001. Then, stereotypically masculine words were rated as less feminine than neutral words, *b* = –1.36, SE = .08, *t*(322.3) = –17.40, *p* < .001 (see Fig 10). Moreover, the analysis also showed a marginally significant Gender × Word type interaction, *b* = .39, SE = .20, *t*(147.1) = 1.93, *p* = .056. *Post-hoc* comparisons revealed that while there were no between-gender differences in the femininity ratings of neutral words, *b* = .05, SE = .04, *t*(144.5) = 1.25, *p* = .213, and stereotypically masculine words, *b* = –.16, SE = .11, *t*(150.8) = –1.41, *p* = .160, stereotypically feminine words were rated as more feminine by females than by males, *b* = .24, SE = .11, *t*(148.2) = 2.14, *p* = .035 (see Fig 11). The reported effects for the English items were adjusted for word valence–a significant predictor of the femininity ratings, *b* = .09, SE = .02, *t*(237.51) = 4.63, *p* < .001. All means with 95% confidence intervals are provided in Table 10.

**Table 10:** Adjusted means (with 95% confidence intervals) of the femininity and masculinity ratings for the English stereotypically feminine, neutral, and stereotypically masculine words.

	Stereotypically feminine words (3 526 observations)	Stereotypically masculine words (3 528 observations)	Stereotypically neutral words (7 092 observations)
Mean femininity ratings	5.42 [5.28, 5.56]	2.65 [2.51, 2.79]	4.01 [3.94, 4.08]
*Females*	5.52 [5.34, 5.70]	2.57 [2.39, 2.75]	4.04 [3.95, 4.12]
*Males*	5.28 [5.11, 5.46]	2.73 [2.55, 2.90]	3.99 [3.92, 4.07]
Mean masculinity ratings	2.79 [2.69, 2.89]	5.46 [5.36, 5.55]	4.01 [3.94, 4.07]
*Females*	2.69 [2.58, 2.80]	5.63 [5.42, 5.64]	3.99 [3.91, 4.07]
*Males*	2.90 [2.79, 3.01]	5.38 [5.27, 5.49]	4.02 [3.94, 4.10]

As for the masculinity ratings of the English items, the analysis also showed a fixed effect of Word type, whereby stereotypically masculine words were rated as more masculine than both neutral words, *b* = 1.45, SE = .06, *t*(243.5) = 24.50, *p* < .001, and stereotypically feminine words, *b* = –2.66, SE = .07, *t*(235.2) = –37.75, *p* < .001. Then, English feminine words were rated as less masculine than English neutral words, *b* = –1.21, SE = .06, *t*(240.3) = –20.12, *p* < .001 (see Fig 10). Also, the analysis also revealed a Gender × Word type interaction, *b* = –.36, SE = .07, *t*(232.7) = –5.29, *p* < .001; *b* = –.18, SE = .06, *t*(244.6) = –3.00, *p* = .003. *Post-hoc* comparisons showed that while there was no between-gender difference in the masculinity ratings of neutral words, *b* = –.03, SE = .04, *t*(347.4) = –.91, *p* = .362, females rated stereotypically feminine words were rated as less masculine, *b* = –.22, SE = .05, *t*(812.3) = –4.49, *p* < .001, and stereotypically masculine words as more masculine, *b* = .15, SE = .05, *t*(817.2) = 3.06, *p* = .003, relative to males (see Fig 11). The reported effects for the English items were adjusted for two significant predictors of the masculinity ratings: word valence, *b* = –.08, SE = .02, *t*(238.2) = –3.98, *p* < .001, and word frequency, *b* = .10, SE = .04 *t*(238.03) = 2.60, *p* = .010. All means with 95% confidence intervals are provided in Table 10.

#### The role of psycholinguistic variables in word ratings

The analyses were conducted separately for femininity and masculinity word ratings. As for the femininity ratings, the ordinal logistic regression revealed that, holding all other predictor variables constant, the odds of rating a stereotypically feminine item as feminine increased by 1.46 (95% CI [1.29, 1.66]) for a one-unit increase in valence as well as by 1.15 (95% CI [1.09, 1.20]) for a one-unit increase in age of acquisition. Moreover, these odds decreased by 0.81 (95% CI [.71,.92]) for a one-unit increase in frequency, by 0.74 (95% CI [.66,.84]) for a one-unit increase in concreteness as well as by 0.68 (95% CI [.60,.77]) for a one-unit increase in the number of syllables. Second, the odds of rating a stereotypically masculine item as feminine increased by 1.13 (95% CI [1.06, 1.20]) for a one-unit increase in valence, by 1.15 (95% CI [1.02, 1.30]) for a one-unit increase in concreteness, as well as by 1.63 (95% CI [1.32, 2.01]) for a one-unit increase in the number of syllables. Furthermore, these odds decreased by 0.84 (95% CI [.77,.91]) for a one-unit increase in the number of letters. Finally, the odds of rating a neutral item as feminine increased by 1.50 (95% CI [1.40, 1.60]) for a one-unit increase in valence, by 1.21 (95% CI [1.12, 1.29]) for a one-unit increase in concreteness, by 1.10 (95% CI [1.00, 1.21]) for a one-unit increase in the number of syllables as well as by 1.10 (95% CI [1.02, 1.16]) for a one-unit increase in the number of letters. Then, these odds decreased by 0.87 (95% CI [.82,.90]) for a one-unit increase in age of acquisition. The estimates for all significant predictors are provided in Table 11.

**Table 11:** The results of ordinal regression for the English items, showing the associations between the femininity ratings and the psycholinguistic variables.

	Stereotypically feminine words			Stereotypically masculine words			Stereotypically neutral words		
	*b*	95% CI	*z*	*b*	95% CI	*z*	*b*	95% CI	*z*
Frequency	–.21	[–.34,–.08]	**–3.19	–	–	–	–	–	–
Valence	.38	[.25,.51]	***5.83	.12	[.06,.19]	***3.66	.40	[.34,.47]	***11.69
Arousal	–	–	–	–	–	–	–	–	–
Concreteness	–.30	[–.42,–.18]	***–4.88	.14	[.01,.27]	*2.19	.19	[.12,.26]	***5.15
Age of acquisition	.14	[.09,.19]	***5.25	–	–	–	–.15	–[.19,–.10]	***–6.34
Syllables	–.39	[–.52,–.26]	***–6.04	.49	[.28,.70]	***4.60	.10	[.00,.19]	*2.07
Letters	–	–	–	–.18	[–.26,–.10]	***–4.25	.09	[.04,.15]	***3.53

* *p* < .05,

** *p* < .01,

*** *p* < .001.

Note.

**Fig 12:**
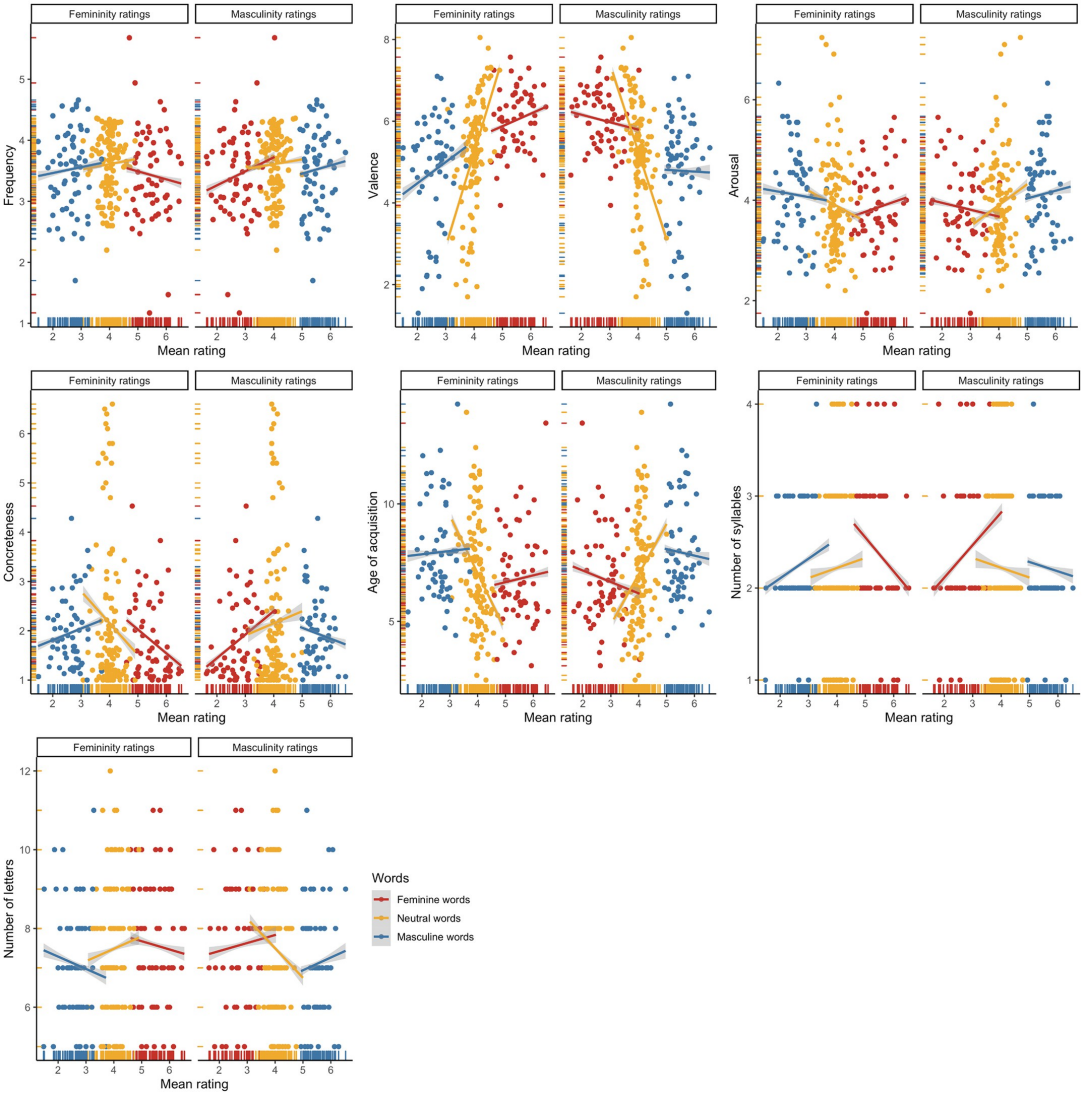
Associations among the mean femininity and masculinity ratings of the English items and the controlled psycholinguistic variables.

As for the masculinity ratings, the ordinal logistic regression revealed that, holding all other predictor variables constant, the odds of rating a stereotypically feminine item as feminine increased by 1.39 (95% CI [1.23, 1.58]) for a one-unit increase in frequency as well as by 1.59 (95% CI [1.40, 1.80]) for a one-unit increase in the number of syllables. Moreover, these odds decreased by 0.69 (95% CI [.61,.78]) for a one-unit increase in valence as well as by 0.90 (95% CI [.86,, 94]) for a one-unit increase in age of acquisition. Second, the odds of rating a stereotypically masculine item as feminine increased by 1.10 (95% CI [1.02, 1.19]) for a one-unit increase in the number of letters. Furthermore, these odds decreased by 0.76 (95% CI [.61,.93]) for a one-unit increase in the number of syllables. Finally, the odds of rating a stereotypically neutral item as feminine increased by 1.17 (95% CI [1.12, 1.22]) for a one-unit increase in age of acquisition. Then, these odds decreased by 0.68 (95% CI [.63,.73]) for a one-unit increase in valence, by 0.79 (95% CI [.74,.85]) for a one-unit increase in concreteness as well as by 0.89 (95% CI [.84,.93]) for a one-unit increase in the number of letters. The estimates for all significant predictors are provided in Table 12. Moreover, the results of the correlational analyses for the English items are provided in Table 13 and Fig 12.

**Table 12:** The results of ordinal regression for the English items, showing the associations between the femininity ratings and the psycholinguistic variables.

	Stereotypically feminine words			Stereotypically masculine words			Stereotypically neutral words		
	*b*	95% CI	*z*	*b*	95% CI	*z*	*b*	95% CI	*z*
Frequency	.33	[.20,.46]	***5.13	–	–	–	–	–	–
Valence	–.37	[–.50,–.25]	***–5.83	–	–	–	–.39	[–.46,–.32]	***–11.29
Arousal	–	–	–	–	–	–	–	–	–
Concreteness	–	–	–	–	–	–	–.23	[–.30,–.16]	***–6.34
Age of acquisition	–.10	[–.15,–.05]	***–4.19	–	–	–	.15	[.11,.20]	***6.78
Syllables	.46	[.34,.59]	***7.24	–.27	[–.47,–.07]	**–2.61	–	–	–
Letters	–	–	–	.10	[.02,.18]	*2.36	–.12	[–.17,–.07]	***–4.60

* *p* < .05,

** *p* < .01,

*** *p* < .001.

Note.

**Table 13:** Pearson correlation coefficients (*r*) showing the associations among the femininity and masculinity scores and the psycholinguistic variables for the English feminine, masculine, and neutral words.

Stereotypically feminine words	1	2	3	4	5	6	7	8
1 Femininity score								
2 Masculinity score	***–.91							
3 Frequency	–	–						
4 Valence	–	–	*.28					
5 Arousal	–	–	**.35	–				
6 Concreteness	*–.29	*.30	–	–	–			
7 Age of acquisition	–	–	–	–	–	**.36		
8 Syllables	–	–	–	–	**–.40	–	–	
9 Letters	–	–	***–.51	–	*–.30	–	–	***.59
**Stereotypically masculine words**								
1 Femininity score								
2 Masculinity score	***–.87							
3 Frequency	–	–						
4 Valence	–	–	**.38					
5 Arousal	–	–	–	***–.49				
6 Concreteness	–	–	–	–	–			
7 Age of acquisition	–	–	**–.38	***–.50	*.34	***.55		
8 Syllables	–	–	–	–	–	–	**.34	
9 Letters	–	–	–	–	–	–	–	***.67
**Stereotypically neutral words**								
1 Femininity score								
2 Masculinity score	***–.87							
3 Frequency	–	–						
4 Valence	***.53	***–.47	–					
5 Arousal	–	–	–	**–.33				
6 Concreteness	–	–	**–.33	***–.49	–			
7 Age of acquisition	**–.32	**.26	***–.42	***–.42	–	***.64		
8 Syllables	–	–	–	–	–	*.19	**.19	
9 Letters	–	–	***–.17	–	–	***.42	***.42	**.29

* *p* < .05,

** *p* < .01,

*** *p* < .001

#### The role of gender role identities in word ratings

The analysis of femininity ratings revealed only a fixed effect of Word type, whereby stereotypically feminine words were rated as more feminine than both neutral words, *b* = –1.47, SE = .08, *t*(324.1) = –17.95, *p* < .001, and stereotypically masculine words, *b* = 2.87, SE = .12, *t*(251.0) = 23.23, *p* < .001. Then, stereotypically masculine words were rated as less feminine than stereotypically neutral words, *b* = 1.40, SE = .08, *t*(323.9) = 17.36, *p* < .001 (see Fig 10 and Table 10).

The analysis of masculinity ratings revealed a fixed effect of Word type, whereby stereotypically masculine words were rated as more masculine than both neutral words, *b* = –1.47, SE = .06, *t*(245.0) = –24.48, *p* < .001, and stereotypically feminine words, *b* = –2.76, SE = .07, *t*(238.8) = –40.13, *p* < .001. Then, stereotypically feminine words were rated as less masculine than stereotypically neutral words, *b* = 1.29, SE = .06, *t*(245.0) = 21.50, *p* < .001 (see Fig 10 and Table 10).

**Fig 13:**
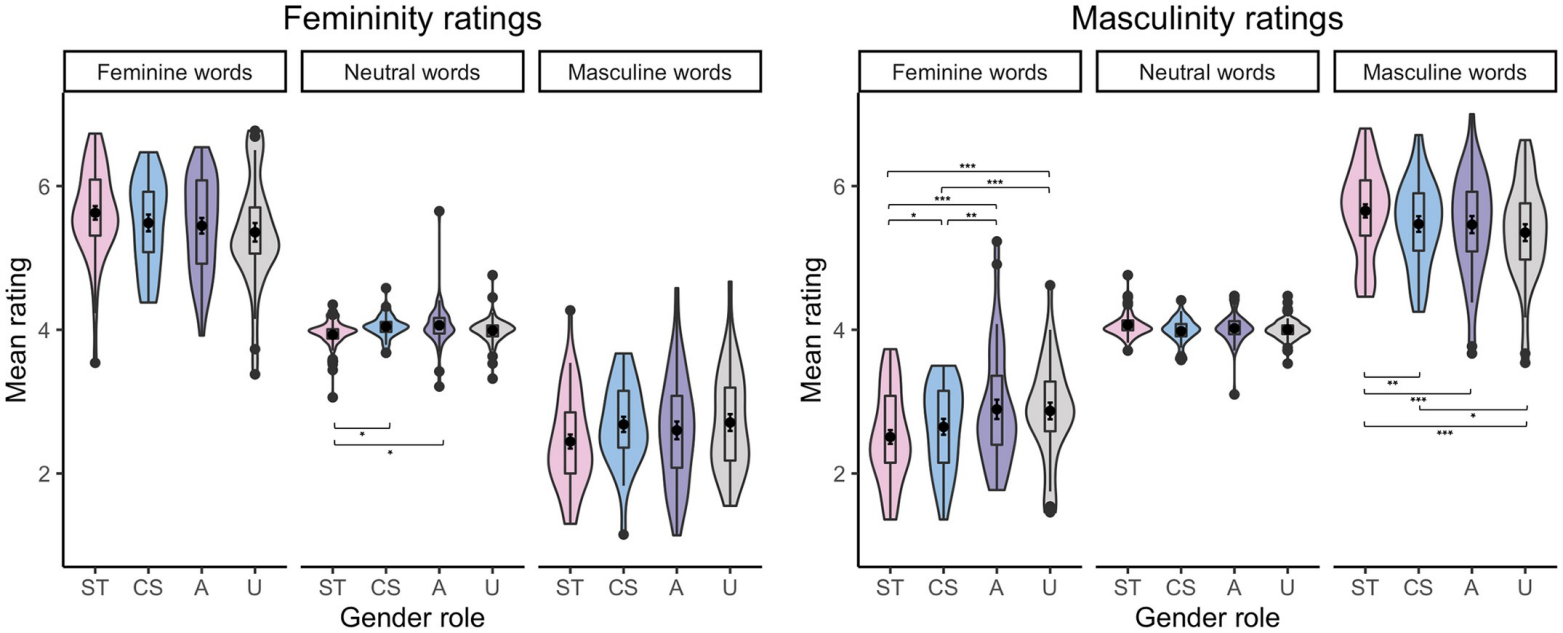
Mean femininity (left) and masculinity (right) ratings of English stereotypically feminine, neutral, and masculine words from participants with the sex-typed (ST), cross-sex (CS), androgynous (A), and undifferentiated (U) gender role identities [* p < .05, ** p < .01, *** p < .001].

The analysis of masculinity ratings also revealed a Word type × Gender role interaction. Regarding stereotypically feminine words, *post-hoc* comparisons indicated that undifferentiated individuals rated them as more masculine than sex-typed individuals, *b* = .41, SE = .07, *t*(942.4) = 6.26, *p <* .001, and than cross-sex individuals, *b* = .25, SE = .07, *t*(902.6) = 3.49, *p <* .001. Similarly, androgynous individuals rated stereotypically feminine words as more masculine than sex-typed individuals, *b* = .40, SE = .07, *t*(832.4) = 6.06, *p <* .001, and than cross-sex individuals, *b* = .24, SE = .07, *t*(810.7) = 3.30, *p =* .001. Finally, cross-sex individuals rated stereotypically feminine words as more masculine than sex-typed individuals, *b* = .16, SE = .07, *t*(905.3) = 2.30, *p <* .001 (see Fig 13 and Table 14).

**Table 14:** Adjusted means (with 95% confidence intervals) of the femininity and masculinity ratings for the English stereotypically feminine, neutral, and stereotypically masculine words, specified by participants’ gender role identities.

	Stereotypically feminine words	Stereotypically neutral words	Stereotypically masculine words
Mean femininity ratings			
*Sex-typed*	5.63 [5.41, 5.85]	3.90 [3.90, 4.08]	2.44 [2.47, 2.95]
*Cross-sex*	5.48 [5.22, 5.75]	4.04 [3.94, 4.15]	2.67 [2.41, 2.93]
*Androgynous*	5.46 [5.22, 5.69]	4.06 [3.96, 4.16]	2.61 [2.37, 2.84]
*Undifferentiated*	5.35 [5.12, 5.59]	3.99 [3.90, 4.08]	2.71 [2.47, 2.95]
Mean masculinity ratings			
*Sex-typed*	2.48 [2.36, 2.60]	4.06 [3.97, 4.15]	5.70 [5.58, 5.82]
*Cross-sex*	2.64 [2.51, 2.78]	3.98 [4.88, 4.08]	5.48 [5.35, 5.62]
*Androgynous*	2.88 [2.75, 3.01]	4.01 [3.91, 4.11]	5.43 [5.30, 5.55]
*Undifferentiated*	2.89 [2.77, 3.02]	4.01 [3.92, 4.10]	5.34 [5.21, 5.47]

Regarding stereotypically masculine words, *post-hoc* comparisons also indicated that undifferentiated individuals rated them as less masculine than sex-typed individuals, *b* = –.36, SE = .07, *t*(937.5) = –5.47, *p <* .001, and than cross-sex individuals, *b* = –.15, SE = .07, *t*(918.6) = –2.02, *p =* .044. Then, androgynous individuals rated stereotypically masculine words as less masculine than sex-typed individuals, *b* = –.27, SE = .07, *t*(829.3) = –4.14, *p <* .001. Finally, cross-sex individuals rated stereotypically masculine words as less masculine than sex-typed individuals, *b* = –.21, SE = .07, *t*(884.9) = –3.06, *p =* .002 (see Fig 13 and Table 14).

### Discussion

The aim of the surveys conducted on English nouns was to examine the degree of gender stereotype load in English as a natural gender language. For the sake of comparability, Study II (involving English items) employed the same procedures as Study I (involving Polish items).

Though we expected the absence of gender stereotype effect in English, the results obtained for the English words yielded the patterns consistent with those observed in Study I. Namely, participants perceived stereotypically feminine nouns as highly feminine, and stereotypically masculine items as high in masculine traits. Furthermore, the study replicated the gender effect observed in Study I, as females’ responses were more in line with gender stereotypes as compared to the ratings provided by male respondents.

Such findings are interesting given that English belongs to a group of languages that are considered more gender-neutral relative to Polish; however, this was not reflected in the obtained results. The findings are therefore indicative of a very robust stereotype load present in single nouns that is independent of language characteristics and manifests itself in both grammatical gender and natural gender languages [73]. Furthermore, the gender effect found in both studies strongly points to potential differences in how males and females perceive gender stereotype, with the effect being independent of the language system.

Similar to Study I, Study II showed that undifferentiated individuals were more neutral in the assessment of femininity and masculinity of words relative to those occupying sex-typed and cross-sex typed gender roles. However, in Study II we also observed that androgynous individuals were generally no different from undifferentiated individuals in their assessment of words indexing femininity and masculinity. This result suggests that having the schemas of both genders (as is in the case of androgynous individuals) decreases participants’ sensitivity to masculine and feminine attributes, indicating that not only undifferentiated but also androgynous individuals might be less sensitive to gender stereotypes. Finally, Study II showed that sex-typed individuals were most sensitive to gender stereotypical features, which is consistent with the gender schema theory positing that sex-typed individuals are more predisposed to apply gender schemas to process sex-typed information [50].

#### Between-language comparison: Grammatical gender (Polish) vs. natural gender (English) language

In order to address the question of whether a language system (i.e., grammatical gender vs. natural gender languages) modulates the gender stereotype load in single nouns, we analyzed the femininity and masculinity ratings using LMMs with Language as a between-subject factor. The analysis of the femininity ratings showed no significant fixed effect of Language, *b* = .10, SE = .06, *t*(595.1) = 1.62, *p* = .106, no Language × Gender interaction, *b* = .07, SE = .06, *t*(304.8) = 1.07, *p* = .286, and no Language × Word type interaction, *b* = –.07, SE = .12, *t*(656.5) = –.57, *p* = .567. Similarly, the analysis of the masculinity ratings revealed no significant fixed effect of Language, *b* = –.06, SE = .05, *t*(526.8) = –1.15, *p* = .250, no Language × Gender interaction, *b* = –.03, SE = .05, *t*(328.3) = –.61, *p* = .543, and no Language × Word type interaction, *b* = –.02, SE = .11, *t*(646.5) = –.18, *p* = .856.

## General discussion

The current contribution presents ratings for stereotypically feminine, stereotypically masculine, and stereotypically neutral (control) Polish (Study I) and English (Study II) nouns. Besides providing gender stereotype norms, the two studies uncover the role of a language system (grammatical gender vs. natural gender language) in the degree of the stereotypical load single nouns carry, and show how respondents’ gender and gender schema (as reflected in Bem Sex-Role Inventory scores [57]) modulate the sensitivity to gender stereotypical attributes.

First of all, our findings from both studies confirmed that the single nouns included in our database are well-selected to illustrate the degree of gender stereotypes assigned to single words. Both Polish and English stereotypical gender and neutral nouns were assessed in line with the categories ascribed to them, showing that participants perceived stereotypically feminine nouns as high in femininity and low in masculinity traits, and stereotypically masculine items as high in masculinity and low in femininity traits. At the same time, stereotypically neutral (control) nouns were consistently perceived as neither feminine nor masculine. These results therefore suggest that exposure to a single lexical item that does not explicitly denote a woman or a man is sufficient to activate gender stereotypes, therefore pointing to a high degree of stereotype load present in both grammatical gender and natural gender languages.

Importantly, the present studies revealed that language system does not necessarily modulate the perception of nouns. Namely, since single words were interpreted as either feminine or masculine in both Study I and Study II, they seem to encode gender stereotypes and assumptions in both grammatical gender (i.e., Polish) and a natural gender (i.e., English) languages. Consequently, the grammatical gender effect might be less relevant than it has previously been concluded [10, 32, 33]. Yet, grammatical gender may still act as an additional factor impacting the perception of single nouns as gendered in accordance with their grammatical gender marking, as revealed in the interaction between grammatical gender and word ratings observed in Study I. In this sense, language as a system may enhance its users’ perception of nouns as more feminine or more masculine. More globally, our results indicate that, despite enormous social and cultural changes, the world continues to be perceived in binary terms with very little room left for non-gendered interpretation.

To provide more thorough insights into the role of grammatical gender, we conducted an additional analysis on Polish items specifically, since these were grammatically-marked and thus included both grammatically feminine and masculine nouns. The results pointed to a grammatical gender effect on the stereotypicality ratings of only stereotypically feminine words, which were perceived as more feminine when they were grammatically feminine and as more masculine when they were grammatically masculine. Such an effect might be due to the fact that stereotypically feminine concepts are often assumed to be more fluid in terms of gender perception [74, 75]. Such increased fluidity of perception around femininity might consequently lead language users to be more sensitive to linguistic cues (like grammatical gender) when evaluating femininity. Potentially, this might stem from the fact that many languages, including Polish, historically reflect male dominance, with masculine grammatical forms often being the “default” [76–78]. Because of this, when a stereotypically feminine word is assigned a masculine grammatical form, it may gain a sense of increased power or dominance, making it seem more masculine. In contrast, grammatically feminine forms might not have the same reinforcing power, as femininity is viewed as subordinate in many languages.

Furthermore, the results showed an effect of participants’ gender, whereby females rated the stimuli as more in line with gender stereotypes relative to males in both languages. Such higher stereotypical ratings given by females might have reflected a stronger internalization of gender stereotypes in females relative to males. A more robust representation of gender stereotypes in females might emanate from the substantial impact gender stereotyping has on females, irrespective of their position or status [45–47]. Due to a continuous exposure to gender stereotypes, females might be more sensitive to detecting stereotypical patterns and associations. As a result of it, they are more likely to perceive gender differences as greater than males (e.g., [42, 43]), which can explain the more stereotype-consistent assessment of the lexical items given by females than males. Complementarily to this interpretation, recent research has suggested that females tend to characterize themselves in more gender stereotypical terms, which is especially evident in their undermined leadership competence and assertiveness self-evaluation [48]. The present results might therefore indicate that females might not have separated themselves from gender stereotypical constraints, and they may still strongly adhere to traditional gender roles, despite some societal changes towards gender equality that are currently being introduced worldwide.

Interestingly, the perception of stereotypical gender features of the nouns was affected by participants’ stereotypically feminine or masculine traits–their gender role measured using BSRI [57]. Namely, based on the gender schema theory [50], we assumed that sex-typed individuals (i.e., females with high levels of feminine traits and low levels of masculine traits, and males with high levels of masculine traits and low levels of feminine traits) would showcase the most robust sensitivity to gender stereotypical features of single words. In line with our expectations, such a pattern was reflected in the present results: sex-typed individuals in Study II showed the most pronounced sensitivity to stereotypically feminine and masculine attributes. These results provide support to the gender schema theory [50], which postulates that sex-typed individuals strongly adhere to their expected male and female gender roles and are more predisposed to use gender schemas to organize and process sex-typed information about themselves and others. This might have consequently made them more sensitive to stereotypical features encoded within the presented nouns.

Furthermore, the results observed in both Study I and Study II revealed a decreased sensitivity to gender stereotypes among undifferentiated individuals (i.e., those that display low levels of both feminine and masculine features). Such findings align with arguments postulated within the self-schema theory [79]. According to this theory, individuals who incorporate feminine and/or masculine attributes into their self-concepts (i.e., those that are either sex-typed, cross-sex-typed, or androgynous) are termed schematic with respect to their gender, while those who have not developed a self-schema reflecting stereotypical aspects of masculinity or femininity are referred to as aschematic [79], and are characterized by an undifferentiated gender schema [80]. The theory further speculates that schematic individuals pay more attention to gender-related attributes than aschematic ones [79]. Following this line of argumentation, we interpret the present findings as suggestive of a somewhat insufficient development of self-schema among aschematic (undifferentiated) participants, which might have consequently attenuated their internalization of stereotypically male and female attributes, making them less sensitive to gender stereotypes encoded within single nouns.

At the same time, the results observed in Study II, where a decreased sensitivity to gender stereotypes was observed among not only undifferentiated but also androgynous individuals, seem not to be in line with other assumptions postulated within the self-schema theory [79] and the gender schema theory [50]. Namely, contrary to what the two theories posit, androgynous individuals, who display high levels of both masculine and feminine traits, were similarly insensitive to gender features as undifferentiated participants. A reduced sensitivity to gender attributes among androgynous individuals might potentially reflect their development of schemas of both genders, as a result of which they may be able to attend to some masculine features within stereotypically feminine words and vice versa, consequently making them less sensitive to the stereotypically expected gender attributes. Overall, the present results confirm that gender role might determine the perception of words’ masculinity and femininity, irrespective of whether the language individuals use is gender-neutral or not.

Finally, we ran exploratory analyses to uncover a potential relationship between word valence and word masculinity/femininity associations, whose results revealed that positively-valenced items were associated with more feminine features, while negatively-valenced words with more masculine traits. Such a negative correlation between positive valence and masculinity is in line with previous research showing that words rated as masculine are more likely to be perceived as negative in valence [55, 56]. This effect might result from the fact that though both stereotypically male agency and stereotypically female communion reflect socially positive traits, communion has been indicated to be more desirable relative to agency [81]. Interestingly, a highly positive perception of communion as a stereotypically feminine characteristic contrasts with a lower status of females in society, which might consequently imply that communal traits, despite being highly desirable, continue to be less relevant as far as positions of power and leaderships are concerned [56, 82]. Such an interpretation seems to be in line with the strong link between valence and the femininity–masculinity dimension that has been previously observed in studies on political discourse, indicating that masculine language is associated with increased negativity and dominance [55]. More research is, nonetheless, needed to further investigate the interplay between word valence and gender stereotypes encoded in single nouns.

Importantly, it needs to be noted that the present research employed survey methodology, which measured participants’ conscious perception of the presented stimuli. Consequently, unlike electrophysiological methods (e.g., electroencephalography) that provide direct insights into how the brain processes stereotype knowledge, survey research might not be sensitive enough to uncover highly automatic and mostly subconscious mechanisms governing stereotype activation. Furthermore, given that the completion of the surveys was self-paced, participants might have employed various strategies when performing the task. For instance, they might have consciously decided to respond in a more non-stereotypical way, in line with their social desirability bias [83, 84]. Future research should therefore investigate whether and to what extent the results observed in the present studies might also be elicited in experimental research examining more implicit gender stereotype perception. Future electrophysiological (EEG) studies should also explore the interplay between grammatical and stereotypical gender, examining how these factors might modulate different stages of language processing. Such studies could determine whether and at what point grammatical gender may override stereotypical gender. By capturing the dynamics of how these types of gender information are processed, EEG research could clarify whether and how stereotypical gender influences gender processing, even when clear grammatical cues are present.

Future studies should also focus on testing languages from cultures that differ in how they conceptualize gender stereotypes. Since femininity and masculinity are cultural constructs ([85]; see also the cultural moderation of gender stereotypes hypothesis; [86]), gender stereotypes vary according to the core cultural values of a given nation [87, 88]. For instance, in collectivistic cultures that value communality and emphasize social connectedness over agency [89, 90], men are stereotyped as collectivistic (i.e., other-oriented). Conversely, in individualistic cultures, which prioritize agency, autonomy, and self-assertion, men are stereotyped as individualistic (i.e., self-oriented) [91–96]. Cuddy et al. [86] demonstrated that Americans, representing an individualistic culture, rate men as less collectivistic than women, while Koreans, representing a collectivistic culture, rate men as more collectivistic than women. Given that the present study tested Polish and English speakers, with both cultures characterized by individualism and short-term orientation [89], more research is needed to investigate the interplay between language, culture, and the perception of gender stereotypes.

## Conclusion

The present contribution shows that gender stereotypical features are strongly internalized in single nouns, even though these do not explicitly denote any male of female referents. Importantly, the current results showed that such an effect was present irrespective of the language system and was observed for both grammatical gender (i.e., Polish) and natural gender (i.e., English) languages, thus suggesting that single words themselves may already showcase a gendered component regardless of grammatical gender. Furthermore, the results yielded a stronger internalization of gender stereotypes among females than males as well as among sex-typed individuals, thus pointing to the crucial role of gender and gender schema in the sensitivity to gender stereotypical attributes. Finally, we observed a negative correlation between word positive valence and stereotypical masculinity, whereby stereotypically masculine nouns were more negatively-valenced, therefore indicating that masculinity might be associated with increased negativity. All in all, the present contribution aims to broaden researchers’ stimulus choices and allow for consistency across different laboratories and research projects on gender stereotype processing. The adaptation of this database to other languages or cultures could also enable a cross-cultural comparison of empirical findings on stereotype processing.
